# Validity of the Kessler Psychological Distress scale in Brazilian higher education students *

**DOI:** 10.1590/1518-8345.7073.4254

**Published:** 2024-11-22

**Authors:** Jaqueline Galdino Albuquerque Perrelli, Gabriel Vinícius Souza de Vasconcelos, Jéssica Rodrigues Correia e Sá, Pollyanna Fausta Pimentel de Medeiros, Roberta Uchôa, Zila Sanchez

**Affiliations:** ^1^ Universidade Federal de São Paulo, Departamento de Medicina Preventiva, São Paulo, SP, Brazil.; ^2^ Universidade Federal de Pernambuco, Departamento de Enfermagem, Recife, PE, Brazil.; ^3^ Universidade Federal de Pernambuco, Departamento de Serviço Social, Recife, PE, Brazil.; ^4^ Scholarship holder at the Conselho Nacional de Desenvolvimento Científico e Tecnológico (CNPq), Brazil.; ^5^ Universidade Federal de Pernambuco, Recife, PE, Brazil.; ^6^ Faculdade de Medicina de Olinda, Unidade Curricular Integração Academia, Serviço e Comunidade, Olinda, PE, Brazil.; ^7^ Centro Universitário Maurício de Nassau, Graças, Recife, PE, Brazil.

**Keywords:** Stress Psychological, Validation Study, Students, Mental Disorders, Higher Education, Reproducibility of Results

## Abstract

to evaluate of the validity of the Brazilian version of the Kessler Psychological Distress Scale, based on its internal structure, concurrent validity, and predictive validity, for the screening of psychological distress among higher education students.

methodological study with 1,034 participants, using the Kessler Psychological Distress Scale as well as the Self-Reporting Questionnaire. An analysis of the internal structure was conducted using a two-factor confirmatory factor analysis, which evaluated fit indices and hierarchical omega reliability coefficients. A Pearson’s correlation test was used to assess concurrent validity, while sensitivity, specificity, areas under the ROC curve and 95% confidence intervals were used to assess predictive validity.

the bifactor model demonstrated excellent fit indices (CFI=1.000; TLI=0.999; SRMR=0.019; RMSEA=0.028; 95%CI: 0.015 - 0.041) as well as high reliability (ωH=0.886). It was observed that there was a strong correlation between the K10 and the SRQ (r=0.813; 95%CI: 0.784 - 0.837). The ideal cut-off point for screening was identified as being higher than 21, with a sensitivity of 85.2% and a specificity of 82.9%.

the structure composed of a general factor, psychological distress, demonstrated a high level of reliability. The scale demonstrated concurrent and predictive validity for the assessment of psychological distress among university students.

## Highlights

**(1)** Validity based on internal structure demonstrated a general factor structure.

**(2)** High reliability of the overall factor structure.

**(3)** Convergent validity of the scale with the SRQ-20 as the gold standard.

**(4)** Predictive validity for psychological distress screening.

**(5)** High sensitivity and specificity for cut-off point greater than 21 points.

## Introduction

 Academic training in higher education is marked by a series of personal, social, academic and institutional challenges. Although this phase offers enriching experiences, it is also a period in which episodes of psychological distress can be intensified, especially among young people. These episodes often stem from factors such as family separation, the need to establish new social relationships, increased autonomy and responsibility, high levels of stress and sleep problems, pressure to be competitive, and difficulties in developing the skills and competencies essential to academic life ^(^ 1 ^-^ 3 ^)^ . 

 It is estimated that around 15% of adults of working age, including higher education students, face or will face mental disorders or episodes of psychological distress at some point in their lives ^(^ 4 ^)^ . Among these, Common Mental Disorders (CMD), such as anxiety and depression, are particularly prevalent. Symptoms range from mild to severe and include changes in mood, thinking, behavior and physical health that affect functioning and quality of life, as well as being associated with a higher risk of suicide ^(^ 5 ^-^ 6 ^)^ . 

 Systematic reviews have highlighted the high prevalence of anxiety, depression, suicidal behavior, eating disorders, post-traumatic stress and sleep disorders among university students, not only in developed countries ^(^ 7 ^-^ 8 ^)^ , but also in low- and middle-income countries ^(^ 9 ^)^ , where more than 80% of the global burden of depression and anxiety is concentrated ^(^ 9 ^)^ . In Brazil, the situation is no different, with high prevalence rates for anxiety (37.7%), depression (28.5%) and suicidal behavior (9.1%) ^(^ 10 ^)^ . However, there is a significant gap in Brazilian national surveys with representative samples of higher education students, especially in the north and northeast regions, which face greater social inequalities ^(^ 10 ^)^ . 

 The promotion of mental health and well-being is included in target 3.4 of the United Nations (UN) Sustainable Development Goals (SDGs), reinforcing the global need to tackle these challenges in an integrated and inclusive manner ^(^ 11 ^)^ . This global context reinforces the importance of screening for mental disorders in students entering higher education, particularly those located in regions with pronounced social inequalities. These conditions not only favor the development of mental disorders ^(^ 12 ^)^ , but they also underline the need for studies to support the implementation of programs aimed at preventing psychological suffering and promoting mental health ^(^ 13 ^)^ , especially in the context of higher education. 

 However, more than estimating the prevalence of the phenomenon, it is necessary to choose the most appropriate tool to provide reliable data on the scenario in question. From this perspective, among the instruments recommended by the World Health Organization (WHO) to screen for mental disorders, especially anxiety, depression and somatic symptoms, the Kessler Psychological Stress Scale (K10) stands out ^(^ 14 ^-^ 16 ^)^ e o Self Reporting Questionnaire (SRQ-20) ^(^ 17 ^-^ 18 ^)^ . These instruments are preferable due to their ease of application and adequate psychometric properties, as shown in previous studies ^(^ 14 ^-^ 16 ^,^ 18 ^-^ 31 ^)^ . 

 This study focuses on investigating the psychometric properties of the Brazilian version of the K10 in the context of higher education. This is a psychological distress screening scale made up of ten items, which represent symptoms of anxiety and depression ^(^ 14 ^-^ 16 ^)^ . Its original English version showed a single-factor structure with high internal consistency (α=0.93) ^(^ 14 ^)^ , as well as predictive validity for screening non-specific psychological distress ^(^ 15 ^)^ . 

 Several international studies have confirmed the reliability of the K10 in different populations and cultures, including Portuguese adults (α=0.91) ^(^ 22 ^)^ , older adults (α=0,84) ^(^ 20 ^)^ and Brazilian adults (α=0,87; α=0,93) ^(^ 19 ^,^ 21 ^)^ , Indonesian adolescents (α=0,89) ^(^ 23 ^)^ , Palestinian social workers (α=0,88) ^(^ 24 ^)^ , primary health care users in Iran (α=0,87) ^(^ 25 ^)^ , general medicine outpatients and caregivers in Uganda (α=0,86) ^(^ 26 ^)^ and in South Africa (α=0,84; ω=0,88) ^(^ 27 ^)^ , aboriginal peoples (α=0,88; α=0,87; α=0,84) ^(^ 28 ^)^ , Australian clinical samples (α=0,93) ^(^ 14 ^,^ 29 ^)^ , as well as Dutch, Turkish and Moroccan adults (α=0,93) ^(^ 30 ^)^ and Americans (α=0,89; α=0,93) ^(^ 31 ^)^ . With regard to the factor structure of the K10, previous research has identified single-factor models ^(^ 15 ^,^ 21 ^,^ 26 ^,^ 28 ^-^ 30 ^)^ , two correlated factors ^(^ 29 ^,^ 31 ^)^ and bifactorial ^(^ 19 ^)^ with two correlated factors, in different contexts and populations. 

 The K10 is translated into several languages, including Brazilian Portuguese ^(^ 32 ^)^ . However, in Brazil, only three studies ^(^ 19 ^-^ 21 ^)^ focused on validity evidence, concentrating on older adults samples ^(^ 20 ^)^ and adults ^(^ 19 ^,^ 21 ^)^ , leaving a gap as to its applicability among university students. Thus, there is a lack of data on the validity of the Brazilian version of the K10 ^(^ 32 ^)^ , especially in relation to its ability to track psychological distress among young people and in the context of higher education. A study of this type is important in that it indicates whether an instrument can adequately measure the construct it is intended to assess, in a particular context and for a particular group. It is important to conduct studies of this type in order to demonstrate that the instrument is capable of measuring the construct it is designed to measure, within a given context and for a particular group of individuals. A poorly structured instrument can lead to erroneous results. In order to ensure the validity of a scale’s internal structure, it is essential to investigate its validity before using it in research or evaluation ^(^ 33 ^)^ . 

 According to Standards for Educational And Psychological Testing ^(^ 34 ^)^ , validity is not a property of a scale, but of the instrument’s scores, so that a continuous source of validity evidence is needed to consider an instrument valid in a given context. In this sense, it is understood that further studies are needed to confirm and/or add new evidence of the validity of the K10 in the Brazilian context. Thus, the aim of this study was to evaluate the validity based on the internal structure, concurrent validity, and predictive validity of the Brazilian version of the Kessler Psychological Distress Scale, for screening psychological distress in higher education students. 

## Method

### Type of study

This is a methodological study with a quantitative approach.

### Setting and period of data collection

Data were collected from April to December 2019 in two Federal Higher Education Institutions (IFES) located in the northeast region of Brazil, a region with a high economic and social vulnerability. There is one of these institutions located in an inland region.

### Population, sample and eligibility criteria

 Participants in the study were higher education students enrolled in face-to-face courses at the research sites of IFES. The sample estimate was calculated using an online calculator ^(^ 35 ^)^ , considering the use of Structural Equation Modeling, using the following parameters ^(^ 36 ^)^ : *a priori* effect size: 0.10; test power: 0.80; number of latent variables: 2; number of observed variables: 10; and probability level: 0.05. This resulted in an estimate of 947 participants. 

To select the sample, the cluster sampling technique was used, with a simple draw of classrooms using Excel software and the Random command, based on a list provided by the institution’s enrollment department, containing the code of each class and the respective course. Once the classrooms had been drawn, the students were selected based on the following inclusion criteria: being aged 18 or over and being in class at the time previously agreed with the teacher for data collection.

Students who reported having clinical problems, such as pain or a mental health crisis, which could prevent them from taking part were excluded from the study. This exclusion criterion was assessed by means of the participant’s report, immediately after the objectives of the research had been explained and the instructions on how to fill in the instruments had been given. If the student reported any clinical condition that prevented them from taking part, the team took up the demand and arranged for them to be referred to the institutions’ psychology service.

In addition to instruments with missing data or erasures in the age variable, instruments with only sociodemographic data, and instruments with an affirmative response to the distracting question were also excluded. This study utilized a distractor question to minimize response bias and related it to the use of a fictitious psychoactive substance. As a result of applying these eligibility criteria, the final sample for this study consisted of 1,034 students, although the initial estimate predicted 947 participants.

### Data collection procedures

Initially, the health team received training on the topic and on the data collection instruments. There were lecturers from the Nursing and Social Work courses, undergraduate students in Social Work and Nursing, as well as other higher education professionals (psychologist, social worker, nurse, and pedagogue).

Following that, the course coordinators were contacted in order to clarify the research, inform the teachers that they would need to give up their classes, and to schedule the data collection. Based on a list of classroom numbers, subjects, class shifts, and courses, the classrooms participating in the study were randomly selected using a simple random selection method.

Potential participants were invited to take part in the study and informed about the voluntary nature of their participation, the objectives of the study, the possible risks and benefits, as well as receiving detailed instructions about the instruments, the average time needed to complete them (60 minutes) and the importance of completing them individually. Data collection took place in the classroom, on days and at times previously agreed with the teacher and the coordinator of the corresponding course.

### Data collection instruments

 The data collection instrument consisted of sociodemographic characterization variables (age, gender, sexual orientation, religion, marital status, number of children, family income, self-reported race/skin color and with whom the student lived), K10 ^(^ 14 ^,^ 32 ^)^ and SRQ-20 ^(^ 17 ^-^ 18 ^)^ . The K10 is self-administered and consists of ten items related to the anxious and depressive symptoms that a person has experienced in the most recent 30-day period. These symptoms together represent psychological distress ^(^ 14 ^)^ . Each item is scored on a five-point Likert scale (1 - Every day; 2 - Most days; 3 - Some days; 4 - Few days; 5 - No days). To calculate the total scores, the five-point scale must first be inverted and then the answers added together. The possible values range from 10 to 50 ^(^ 14 ^)^ . The original English version of the scale showed high internal consistency (α=0.93) ^(^ 14 ^)^ . As for the cut-off points, a score greater than 17 showed a sensitivity of 81.1% and specificity of 83.0% for identifying psychological distress ^(^ 37 ^)^ . 

 In this study, three validity dimensions of the Brazilian Portuguese version of the K10 were investigated: validity based on internal structure, concurrent validity, and predictive validity. Validity based on internal structure assesses the extent to which the correlations between items reflect the structure of the construct that the instrument is intended to measure, and is commonly explored through factor analysis ^(^ 34 ^)^ . 

 Concurrent validity seeks to determine the association between the K10 and other instruments that measure the same construct, while predictive validity focuses on whether K10 scores can predict participants’ performance on future tests or behaviors, specifically the occurrence of psychological distress ^(^ 34 ^)^ . These validities were examined by comparing the K10 with the SRQ-20, the latter adopted as the gold standard. 

 The SRQ-20 is a self-report questionnaire comprising 20 items relating to non-psychotic symptoms (somatic, depressive, and anxious), with dichotomous responses (yes/no). A final score is calculated as the sum of all affirmative responses, ranging from zero to twenty points ^(^ 17 ^-^ 18 ^)^ . To detect a suggestive condition of CMD, in the Brazilian version, a cut-off point above 7.0 points was established with strong evidence of criterion validity (sensitivity = 86.33%, specificity = 89.31%). Further, this particular version has demonstrated high reliability, as indicated by the Cronbach’s alpha coefficient (α=0.86) ^(^ 18 ^)^ . 

### Data analysis

 Data was organized in an Excel spreadsheet and analyzed using JASP - version 0.17.2.1 and MedCalc - free trial version. The sample was characterized by frequency distributions and measures of central tendency. The validity evidence derived from the internal structure of the K10 was verified using Structural Equation Modeling and Bifactor Confirmatory Factor Analysis (Bifactor CFA), and compared to previously reported single factor models ^(^ 15 ^,^ 21 ^,^ 26 ^,^ 28 ^-^ 30 ^)^ , two correlated factors ^(^ 29 ^,^ 31 ^)^ and bifactorial models ^(^ 19 ^)^ of K10. 

 A related bifactor and hierarchical model has been utilized to conceptualize, study and diagnose psychopathologies, and makes it possible to determine if a given observable variable can be adequately explained either by a general or specific dimension, based on a comparison of several models ^(^ 38 ^-^ 41 ^)^ . 

 In the Bifactor CFA, the latent variables were orthogonally positioned so that their explained variances with respect to each observable variable did not overlap ^(^ 38 ^-^ 39 ^)^ . In addition, the Robust Diagonally Weighted Least Squares (RDWLS) estimation method was used, which is suited to categorical data ^(^ 42 ^-^ 43 ^)^ . The adequacy of the model was assessed using the fit indices: χ² (chi-square), Comparative Fit Index (CFI), Tucker-Lewis Index (TLI), Standardized Root Mean Square Residual (SRMR) and Root Mean Square Error of Approximation (RMSEA). The ratio between the χ² value and the model’s degrees of freedom (gl) must be less than 3.0 to indicate a good model fit, while values above 5.0 indicate an inadequate fit. CFI and TLI values should be greater than or equal to 0.90, with values above 0.95 being preferred. The SRMR should be a maximum of 0.08, and the RMSEA value should be less than or equal to 0.06 or a maximum of 0.08, with a confidence interval (upper limit) less than or equal to 0.10 ^(^ 44 ^)^ . 

 In addition to the fit indices noted above, the standardized factor loadings in each of the three models tested were also evaluated, with those with values above 0.40 being more significant. These values are relevant since they indicate a significant contribution by the items to the factor in question. For each factor, the Average Variance Extracted (AVE) was also analyzed. A variable’s AVE quantifies the proportion of variance captured by its items in relation to the variance attributed to measurement errors or single variances. In the presence of a high SVA, it is likely that most of the variance of the items is shared with the underlying construct, which reinforces the internal consistency of the factor. In contrast, a low SVA may indicate that the construct items fail to adequately capture the common variance or that there is a significant amount of variance due to measurement errors. Generally, VME values above 0.5 are considered satisfactory, while those below this level may indicate the need to revise or improve the model ^(^ 45 ^)^ . 

 To assess the reliability of the factor structure, the Hierarchical Omega (wH) was used to assess the degree to which an aggregate measure can be explained by a general factor ^(^ 46 ^)^ . A value greater than 0.70 indicates a high level of reliability for this general factor ^(^ 39 ^)^ . Accordingly, the model whose items had the highest standardized factor loadings, highest SEM, best fit, and reliability indices ^(^ 47 ^)^ was considered to be the most suitable to represent the factor structure of the K10. 

 Based on the SRQ-20 as the gold standard, Pearson’s correlation coefficient was calculated along with their respective confidence intervals from bootstrapping resampling to determine the concurrent validity of the K10. Correlations greater than 0.70 indicate adequate correlation ^(^ 34 ^)^ . 

 Finally, predictive validity was evaluated by analyzing the receiver operator characteristic (ROC curve), measures of accuracy (sensitivity and specificity), and their respective 95% Confidence Intervals (CI) in accordance with Clopper-Pearson. If the Area Under The Curve (AUC) is greater than or equal to 0.80, indicating high sensitivity and specificity, the test is adequate for screening a given phenomenon, when a specific cut-off point is considered ^(^ 48 ^)^ . 

### Ethical aspects

The study was approved by the Ethics and Research Committee with Human, under opinion number 2.937.477.

## Results

Age ranged from 18 to 25 years (mean=23.5; standard deviation=7.17), with higher proportions of students in the age groups up to 20 years (n=537; 44.9%) and 21 to 30 years (n=511; 42.7%). There was a similar proportion of men (n=596; 49.8%) and cisgender women (n=581; 48.5%), and 85.8% (n=1,027) identified themselves as heterosexual. A percentage of 68.6% (n 821) reported following some form of religion. The majority reported being single (n=1,034; 86.4%) and having no children (n=1,069; 89.3%). The average family income was R$ 2,847.40. More than half declared themselves to be brown (n=626; 52.3%), and 47.0% (n=563) lived with their mother and father.

 Based on the comparison of CFA models ( Table 1 ), the two-factor correlated and bifactor models were found to have better fit indices. In particular, the bifactor model showed higher values in terms of CFI (1.000), TLI (0.999), SRMR (0.0019) and RMSEA (0.028; CI: 0.015 - 0.041). 

**Table 1 -:** Fit indices of different factor models of the Brazilian Portuguese version of the Kessler Psychological Stress Scale in a sample of higher education students (n = 1,197). Recife, PE, Brazil, 2019

**Models**	χ ^2*^	**gl** ^†^	**CFI** ^‡^	**TLI** ^§^	**SRMR** ^||^	**RMSEA** ^¶^	**IC 90%** ^**^	
1 factor	483,697 ^††^	35	0.992	0.989	0.058	0.111	0.103	0.120
2 factors correlation	166,066 ^††^	34	0.998	0.997	0.036	0.061	0.052	0.071
Bi-factor	45.895 ^‡‡^	25	1.000	0.999	0.019	0.028	0.015	0.041

^*^ χ ^2^ = Chi-square

^†^ gl =Degrees of freedom

^‡^ CFI = Comparative Fit Index

^§^ TLI = Tucker-Lewis Index

^||^ SRMR = Standardized Root Mean Squared Residual

^¶^ RMSEA = Root Mean Square Error of Approximation

^**^ 90%CI = 90% Confidence interval

^††^ p<0,001

^‡‡^ p<0,05

 However, it is necessary to evaluate other measures to determine the most appropriate factor structure, including factor loadings, VME and wH, especially with regard to the bifactor model, which tends to show overfitting, i.e., overestimated fit indices. With regard to factor loadings ( Table 2 ), those associated with the general factor - psychological distress - varied between 0.69 and 0.91. For the specific factors, the variations ranged from 0.22 to 0.90 for the depressive symptoms factor and from -0.08 to 0.89 for the anxious symptoms factor. Items such as 1, 2, 3 and 6 had factor loadings of less than 0.30 on the specific factors, suggesting a variable relationship with the factors and indicating that some items have negative loadings, especially on the anxious symptoms factor. These results suggest that the most appropriate structure for the instrument includes a general factor - psychological distress, given the higher factor loadings observed in the single-factor and two-factor models. 

 The analysis of explained variance ( Table 2 ) shows that the one-factor model was able to explain 53.8% of the variance in the items, which is 3.8% more than the general factor - psychological distress in the two-factor model (50.0%). In contrast, the correlated two-factor model revealed significantly more explained variance in the two factors (depressive symptoms - 50.9%; anxious symptoms - 73.5%) compared to the specific factors in the bifactor model (depressive symptoms - 20.0%; anxious symptoms - 5.0%). 

This shows that the bifactor model explained 75.0% of the total variance of the items, indicating a significant increase in relation to the other models, with the SVA of the general factor significantly higher than that of the specific factors. Therefore, when controlling for the effect of the specific factors and disregarding the number of items, the general factor explained most of the variance in the items. In other words, when adjusting for the effect of the general factor, the internal consistencies of the specific factors are low. This indicates that the variability of the items is mostly explained by the general factor, reducing the relevance of the specific factors in explaining the variance of the items.

As for the reliability of the factor structures (Table 2), the one-factor model and the general factor of the two-factor model had the highest reliability indices, with hierarchical omega of 0.996 and 0.886, respectively. The correlated two-factor models and the bifactor model when considering the specific factors had lower reliability indices, indicating that these models are less accurate. In particular, the bifactor model showed the lowest internal consistency in the specific factors, when controlling for the effect of the general factor.

**Table 2 -:** Standardized factor loadings for the factor models of the Kessler Psychological Stress Scale in a sample of higher education students (n = 1,197). Recife, PE, Brazil, 2019

**Itens**	**Standardized factor loadings**					
	**Traditional Confirmatory Factor Analysis**			**Bifactor Confirmatory Factor Analysis**		
	**Single-factor model**	**Correlated two-factor model**		**General factor**	**Specific factors**	
	PS ^*^	DS ^†^	AS ^‡^	PS ^*^	DS ^†^	AS ^‡^
Item 1	0.73	0.75		0.70	0.22	
Item 2	0.80		0.86	0.86		-0.08
Item 3	0.84		0.89	0.91		-0.23
Item 4	0.82	0.84		0.70	0.47	
Item 5	0.78		0.83	0.83		0.45
Item 6	0.80		0.85	0.83		0.21
Item 7	0.89	0.90		0.73	0.56	
Item 8	0.78	0.80		0.71	0.35	
Item 9	0.88	0.90		0.74	0.52	
Item 10	0.84	0.85		0.69	0.53	
Average Variance Explained (%)	53.8	50.9	73.5	50.0	20.0	5.0
Hierarchical Omega	0.996	0.648	0.460	0.886	0.106	0.016

^*^ PS = Psychological distress

^†^ DS= Depressive symptoms

^‡^ AS= Anxious symptoms

The results obtained by the CFA showed that the bifactor model was superior in representing the structure of the K10 for this sample, as evidenced by several aspects. Firstly, the fit indices of the bifactor model, made up of a general factor - psychological distress - and two specific factors - depressive symptoms and anxious symptoms, were significantly higher. In addition, the factor loadings were higher for the general factor, highlighting its preponderance in the structure of the instrument and indicating high internal consistency. This model also stands out for its ability to explain a greater portion of the variance in the items.

By comparing the two-factor model with the one-factor model, it is clear that the latter has higher factor loadings and satisfactory internal consistency, but its fit indices are inadequate, which gives the two-factor model an advantage. The analysis of the variance explained by both models, when evaluating the overall factor, was similar. However, the SEM of the general factor, together with the specific factors in the two-factor model, shows a higher explained variance than the single-factor model. This comparison reinforces the superiority of the bifactor model, particularly due to its ability to capture a broader dimension of variance, strengthening its applicability and accuracy in analyzing the structure of the K10 for the sample studied.

**Figure 1 -:**
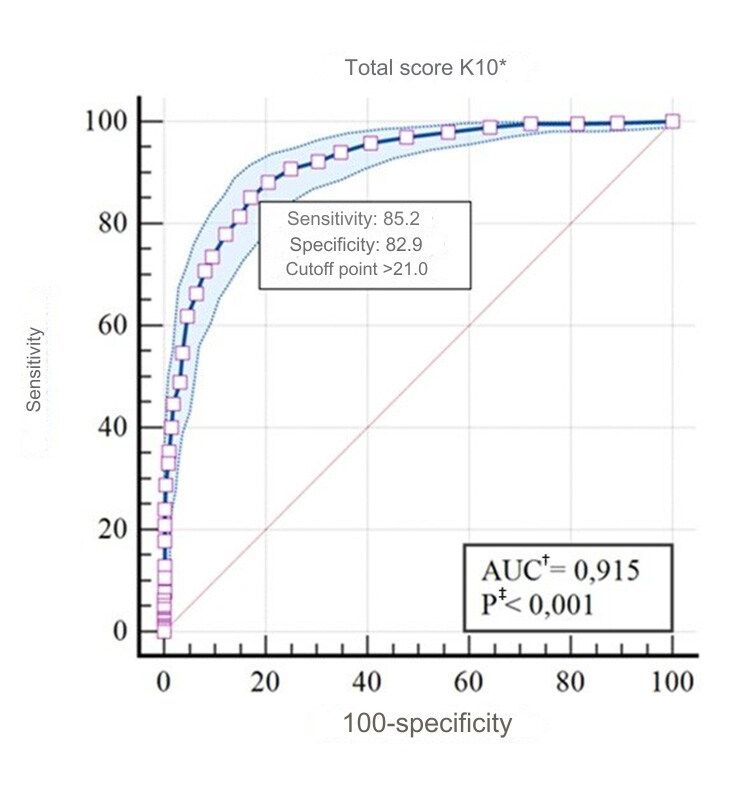
Area under the ROC curve of the Kessler Psychological Stress Scale, Brazilian Portuguese version

 Regarding concurrent validity, there was a strong correlation between the K10 and SRQ-20 scores of r=0.813 (95% CI Bootstrapping: 0.784 - 0.837; r2=0.660; p<0.001), which indicates the concurrent validity of the K10 for screening psychological distress. In terms of predictive validity, the best K10 cut-off point for this screening was a total score greater than 21, with sensitivity of 85.2% (95% CI: 81.6 - 88.3) and specificity of 82.9% (95% CI: 79.9 - 85.6), both high, as well as an excellent AUC value of 0.915 (95% CI Bootstrapping: 0.896 - 0.929; p<0.0001) ( Figure 1 ). 

*K10 = Kessler Psychological Stress Scale; ^†^ AUC= Area Under the Curve; ^‡^ P= p-value

 Thus, based on these results and from this cut-off point, it is possible to correctly screen 85.2% of cases of psychological distress, with a false positive rate of 17.1. Further details can be found in Table 3 . 

**Table 3 -:** Cut-off points of the Kessler Psychological Stress Scale for screening psychological distress in students (n = 1,197). Recife, PE, Brazil, 2019

**Cut-off poin** t	**Sensitivity**	**95%CI** ^*^	**Specificity**	**95%CI** ^*^
≥10	100.00	99.2 - 100.0	0.00	0.0 - 0.5
>10	99.79	98.8 - 100.0	10.72	8.6 - 13.2
>11	99.58	98.5 - 99.9	18.66	15.9 - 21.7
>12	99.58	98.5 - 99.9	27.86	24.6 - 31.3
>13	98.94	97.5 - 99.7	35.79	32.3 - 39.4
>14	97.88	96.1 - 99.0	44.01	40.3 - 47.7
>15	97.03	95.1 - 98.4	52.23	48.5 - 55.9
>16	95.76	93.5 - 97.4	59.33	55.6 - 62.9
>17	94.07	91.5 - 96.0	65.18	61.6 - 68.7
>18	92.16	89.4 - 94.4	69.64	66.1 - 73.0
>19	90.68	87.7 - 93.1	74.93	71.6 - 78.1
>20	88.14	84.9 - 90.9	79.39	76.2 - 82.3
>21	85.17	81.6 - 88.3	82.87	79.9 - 85.6
>22	81.36	77.5 - 84.8	84.96	82.1 - 87.5
>23	77.97	74.0 - 81.6	87.74	85.1 - 90.1
>24	73.52	69.3 - 77.4	90.39	88.0 - 92.4
>25	70.76	66.4 - 74.8	91.92	89.7 - 93.8
>26	66.31	61.9 - 70.6	93.45	91.4 - 95.2
>27	61.86	57.3 - 66.3	95.40	93.6 - 96.8
>28	54.66	50.0 - 59.2	96.24	94.6 - 97.5
>29	48.94	44.3 - 53.6	96.80	95.2 - 98.0
>30	44.70	40.2 - 49.3	98.05	96.8 - 98.9
>31	40.04	35.6 - 44.6	98.47	97.3 - 99.2
>32	35.38	31.1 - 39.9	98.89	97.8 - 99.5
>33	33.05	28.8 - 37.5	99.03	98.0 - 99.6
>34	28.81	24.8 - 33.1	99.58	98.8 - 99.9
>35	23.94	20.2 - 28.1	99.72	99.0 - 100.0
>36	20.97	17.4 - 24.9	99.72	99.0 - 100.0
>37	17.80	14.5 - 21.6	99.72	99.0 - 100.0
>38	12.92	10.0 - 16.3	99.72	99.0 - 100.0
>39	10.59	8.0 - 13.7	99.72	99.0 - 100.0
>40	7.84	5.6 - 10.6	99.72	99.0 - 100.0
>41	6.14	4.2 - 8.7	99.86	99.2 - 100.0
>42	5.08	3.3 - 7.5	99.86	99.2 - 100.0
>43	4.45	2.8 - 6.7	99.86	99.2 - 100.0
>44	2.75	1.5 - 4.7	99.86	99.2 - 100.0
>45	2.33	1.2 - 4.1	99.86	99.2 - 100.0
>46	2.12	1.0 - 3.9	100.00	99.5 - 100.0
>47	1.27	0.5 - 2.7	100.00	99.5 - 100.0
>48	0.85	0.2 - 2.2	100.00	99.5 - 100.0
>49	0.64	0.1 - 1.8	100.00	99.5 - 100.0
>50	0.00	0.0 - 0.8	100.00	99.5 - 100.0

^*^ 95%CI = 95% Confidence Interval of Clopper-Pearson

## Discussion

 This research investigated the validity evidence of the K10, Brazilian Portuguese version, for screening psychological distress in higher education students, based on internal structure, concurrent validity, and predictive validity. In this study, the results, derived from the Bifactor CFA model, showed better fit indices in line with a previous study carried out with a sample of Brazilian adults ^(^ 19 ^)^ . In addition, the general factor of psychological distress showed high internal consistency and explained a significant percentage of the variance, in line with previous research which pointed to a single-factor structure composed of psychological distress ^(^ 15 ^,^ 19 ^,^ 26 ^,^ 28 ^-^ 30 ^)^ . 

 Bifactor models are especially useful when you want to understand not only a global dimension, but also specific variations around that dimension, offering a deeper and more diverse view of the relationships between variables than other Factor Analysis models ^(^ 38 ^-^ 41 ^)^ . This multifaceted approach is essential to deepen our understanding of the structure of psychological suffering and its varied manifestations. 

 From this perspective, the selection and application of K10, as highlighted by some authors ^(^ 19 ^,^ 29 ^)^ , must be closely linked to the specific purpose of the instrument and the profile of the individuals being assessed. In the context of community populations and screening processes, the detection of general psychological distress, to the detriment of specific conditions such as anxiety and depression, is more appropriate. On the other hand, when it comes to assessing a clinical population, differentiating between specific problems, such as anxiety and depression, is more appropriate ^(^ 29 ^)^ , which indicates the K10’s flexibility in meeting different assessment needs. 

 The relevance of this approach is especially significant when considering higher education students, in whom the concomitant presence of anxious and depressive symptoms is notable ^(^ 3 ^,^ 10 ^,^ 49 ^-^ 53 ^)^ . This reality supports the structure of a general psychological distress factor identified in this study. In fact, although this suffering is often conceived, from a two-dimensional perspective, as the coexistence of anxious and depressive symptoms, these conditions act as bidirectional risk factors for each other ^(^ 54 ^-^ 55 ^)^ . While anxiety is associated with fear, nervousness, excessive worry and a feeling of tension, depression is related to sadness, hopelessness, lack of interest or pleasure in daily activities, fatigue and suicidal behavior (either suicidal ideation, attempted or completed suicide) ^(^ 52 ^-^ 53 ^,^ 56 ^)^ . 

 Authors have highlighted the high rate of comorbidity between depression and anxiety, with significant impacts on the functionality of university students ^(^ 57 ^-^ 58 ^)^ . The overlap of these conditions and their risk factors indicate a close relationship between these disorders, suggesting that they should be considered as manifestations of an internalizing psychiatric syndrome, characterized by inward-looking symptoms such as anxiety and depression. This conception has been proposed as beneficial for screening and treatment ^(^ 59 ^)^ . The simultaneous presence of these conditions intensifies psychological suffering and has a negative impact on students’ quality of life ^(^ 57 ^-^ 58 ^)^ . It is therefore crucial to understand this phenomenon in its totality and diversity of symptoms, to facilitate the design and implementation of more effective therapeutic interventions. 

 In terms of concurrent validity, the K10 showed a high correlation with the SRQ-20, one of the most widely used instruments for CMD screening. The K10’s performance in terms of concurrent validity is in line with previous studies that have confirmed this psychometric property ^(^ 14 ^,^ 60 ^)^ . In terms of the construct assessed, both instruments explore similar phenomena, but with their own specificities. The K10, made up of ten items, addresses psychological distress through depressive and anxious symptoms, using a five-point Likert scale for responses ^(^ 14 ^)^ . The SRQ-20, with its 20 items, investigates somatic, depressive and anxiety symptoms associated with CMD, using dichotomous yes/no answers ^(^ 17 ^-^ 18 ^)^ . 

 In terms of the continuum of responses, some studies ^(^ 61 ^-^ 62 ^)^ suggest that the reliability of scores tends to increase as the number of response options increases. However, other researchers ^(^ 63 ^)^ did not observe significant improvements in the psychometric accuracy of instruments that offer more than six response alternatives. They point out that dichotomous scales have limitations in terms of internal consistency when compared to formats that offer a wider range of choices. Therefore, when selecting an assessment instrument, it is advisable to consider not only the diversity of response options, but also ease of application and psychometric properties validated in different samples and previous studies ^(^ 63 ^)^ . 

 The SRQ-20, despite offering only two response options, is widely recognized and recommended by the WHO ^(^ 17 ^)^ due to its simplicity, low cost and good psychometric properties. The K10, also characterized by its ease of use and affordable cost ^(^ 14 ^)^ , has robust psychometric properties, which were reiterated by the results of this study. Therefore, the choice between the SRQ-20 and the K10, although both assess aspects of psychological distress, depends essentially on the specifics that the researcher wishes to explore. If the focus is on detecting somatic symptoms together with depressive and anxious symptoms, the SRQ-20 is more suitable. However, for a more detailed investigation of psychological distress from a broad perspective, covering the variety of depressive and anxious symptoms through a Likert scale, the K10 is the more suitable option. 

 In this study, the K10 Scale stood out as having a higher AUC compared to the values found in previous studies, which recorded AUC of 0.90 ^(^ 16 ^)^ , 0,82 ^(^ 23 ^)^ and 0,86 ^(^ 64 ^)^ . The Brazilian Portuguese version proved to be accurate for screening psychological distress among university students, achieving high sensitivity and specificity rates for scores above 21. The literature suggests that screening instruments should exhibit high rates of sensitivity and specificity at a well-defined cut-off point ^(^ 48 ^)^ . In this study, scores above 21 met these criteria, in line with the findings of studies using the English version of the K10, in which the cut-off point ranged from 18 to 27 points ^(^ 16 ^,^ 23 ^,^ 64 ^)^ . 

Although this study offers promising evidence of the applicability of the Brazilian Portuguese version of the K10 in the context of higher education, it is necessary to consider its limitations, such as the fact that it was carried out in only two HEIs, limiting the generalization of the results to other Brazilian HEIs. A more in-depth examination of the K10’s psychometric properties is therefore suggested, including the application of Item Response Theory (IRT) to samples from different regions of the country, to enable the continuous refinement of this instrument.

Despite these limitations, the global structure of the K10, represented in this study by a general factor - psychological distress, has validity based on internal structure, concurrent and predictive validity, and is established as a reliable instrument for screening psychological distress in higher education students. This tool can make a significant contribution to the design and implementation of health interventions aimed at preventing this condition in the academic context.

## Conclusion

This study provides robust evidence of the validity of the K10, supported by the internal structure and the concurrent and predictive validities, which will help in the screening of psychological distress in higher education students. The Bifactor CFA revealed excellent fit indices for the bifactor model, in which a general factor - psychological distress - explained most of the variance in the K10 items and showed high reliability. In addition, the scale demonstrated concurrent and predictive validity. The K10’s ideal cut-off point for screening psychological distress in these students was identified, a value in line with the results observed in previous investigations with the original version.
