# Supplementary material for: Validity of the Kessler Psychological Distress scale in Brazilian higher education students
Source: Rev Lat Am Enfermagem. 2024 Nov 22;32:e4254. doi: 10.1590/1518-8345.7073.4254 (PMC11654030; doi:10.1590/1518-8345.7073.4254)
Supplement: Translation

# Translation: Validez de la escala de Sufrimiento Psicológico de Kessler en estudiantes brasileños de educación superior *

**DOI:** 10.1590/1518-8345.7073.4253

**Published:** 2024-11-15

**Authors:** Jaqueline Galdino Albuquerque Perrelli, Gabriel Vinícius Souza de Vasconcelos, Jéssica Rodrigues Correia e Sá, Pollyanna Fausta Pimentel de Medeiros, Roberta Uchôa, Zila Sanchez

**Affiliations:** ^1^ Universidade Federal de São Paulo, Departamento de Medicina Preventiva, São Paulo, SP, Brasil.; ^2^ Universidade Federal de Pernambuco, Departamento de Enfermagem, Recife, PE, Brasil.; ^3^ Universidade Federal de Pernambuco, Departamento de Serviço Social, Recife, PE, Brasil.; ^4^ Becario del Conselho Nacional de Desenvolvimento Científico e Tecnológico (CNPq), Brasil.; ^5^ Universidade Federal de Pernambuco, Recife, PE, Brasil.; ^6^ Faculdade de Medicina de Olinda, Unidade Curricular Integração Academia, Serviço e Comunidade, Olinda, PE, Brasil.; ^7^ Centro Universitário Maurício de Nassau, Graças, Recife, PE, Brasil.

**Keywords:** Estrés Psicológico, Estudio de Validación, Estudiantes, Transtornos Mentales, Educación Superior, Reproducibilidad de los Resultados

## Abstract

evaluar la validez basada en la estructura interna, validez concurrente y predictiva de la versión brasileña de la Escala de Sufrimiento Psicológico de Kessler, para el rastreo de sufrimiento psicológico en estudiantes de nivel superior.

estudio metodológico con 1.034 participantes, aplicándose la Escala de Sufrimiento Psicológico de Kessler y el *Self Reporting Questionnaire* . El análisis de la estructura interna fue investigado mediante el Análisis Factorial Confirmatorio bifactorial, evaluándose los índices de ajuste y el coeficiente de fiabilidad omega jerarquizado. La validez concurrente se verificó por el test de correlación de Pearson, mientras para la validez predictiva se utilizaron sensibilidad, especificidad, área bajo la curva ROC e intervalo de confianza del 95%.

el modelo bifactorial presentó excelentes índices de ajuste (CFI=1,000; TLI=0,999; SRMR=0,019; RMSEA=0,028; IC95%: 0,015 – 0,041) y alta fiabilidad (ωH=0,886). Hubo una fuerte correlación significativa entre la K10 y el SRQ (r=0,813; IC95%: 0,784 – 0,837). El punto de corte ideal para el rastreo fue identificado como superior a 21, con una sensibilidad del 85,2% y una especificidad del 82,9%.

la estructura compuesta por un factor general - sufrimiento psicológico presentó alta fiabilidad. La escala mostró validez concurrente y predictiva para el rastreo de sufrimiento psicológico entre estudiantes universitarios de educación superior.

## Destacados

**(1)** Validez basada en la estructura interna demostró estructura de un factor general.

**(2)** Alta fiabilidad de la estructura de un factor general.

**(3)** Validez convergente de la escala teniendo el SRQ-20 como patrón oro.

**(4)** Validez predictiva para el cribado de sufrimiento psicológico.

**(5)** Sensibilidad y especificidad elevadas para punto de corte mayor que 21 puntos.

## Introducción

 La formación académica en la educación superior está marcada por una serie de desafíos de naturaleza personal, social, académica e institucional. Aunque esta fase propicia experiencias enriquecedoras, también es un período en el que los episodios de sufrimiento psicológico pueden intensificarse, especialmente entre los jóvenes. Tales episodios frecuentemente derivan de factores como la separación familiar, la necesidad de establecer nuevas relaciones sociales, el aumento de la autonomía y responsabilidad, altos niveles de estrés y problemas de sueño, presión por la competitividad, y dificultades en el desarrollo de habilidades y competencias esenciales para la vida académica ^(^ 1 ^-^ 3 ^)^ . 

 Se estima que cerca del 15% de los adultos en edad productiva, incluyendo estudiantes de nivel superior, enfrentan o enfrentarán trastornos mentales o episodios de sufrimiento psicológico en algún momento de sus vidas ^(^ 4 ^)^ . Entre estos, los Trastornos Mentales Comunes (TMC), como la ansiedad y la depresión, son particularmente prevalentes. Los síntomas varían de leves a graves e incluyen alteraciones del ánimo, pensamiento, comportamiento y salud física que afectan el funcionamiento y la calidad de vida, además de estar asociados al mayor riesgo de suicidio ^(^ 5 ^-^ 6 ^)^ . 

 Revisiones sistemáticas han destacado la elevada prevalencia de ansiedad, depresión, comportamiento suicida, trastornos alimentarios, estrés postraumático y disturbios del sueño entre universitarios, no solo en países desarrollados ^(^ 7 ^-^ 8 ^)^ , sino también en países de baja y media renta ^(^ 9 ^)^ , donde más del 80% de la carga global de depresión y ansiedad está concentrada ^(^ 9 ^)^ . En Brasil, la situación no es diferente, con altas tasas de prevalencia para ansiedad (37,7%), depresión (28,5%) y comportamiento suicida (9,1%) ^(^ 10 ^)^ . Sin embargo, se nota una laguna significativa de levantamientos nacionales brasileños con muestras representativas de estudiantes de nivel superior, especialmente en las regiones norte y nordeste, que enfrentan mayores desigualdades sociales ^(^ 10 ^)^ . 

 La promoción de la salud mental y del bienestar está presente en el objetivo 3.4 de los Objetivos de Desarrollo Sostenible (ODS) de la Organización de las Naciones Unidas (ONU), reforzando la necesidad global de enfrentar estos desafíos de manera integrada e inclusiva ^(^ 11 ^)^ . Este contexto global refuerza la importancia de rastrear cuadros sugestivos de trastornos mentales en estudiantes que ingresan a la educación superior, particularmente en aquellos situados en regiones con desigualdades sociales pronunciadas. Estas condiciones no solo favorecen el desarrollo de trastornos mentales ^(^ 12 ^)^ , sino que también subrayan la necesidad de estudios que fundamenten la implementación de programas orientados a la prevención del sufrimiento psicológico y a la promoción de la salud mental ^(^ 13 ^)^ , sobre todo en el contexto de la educación superior. 

 Sin embargo, más que estimar la prevalencia del fenómeno, se hace necesario elegir la herramienta más adecuada para proporcionar datos fiables sobre el escenario en cuestión. En esta perspectiva, entre los instrumentos recomendados por la Organización Mundial de la Salud (OMS) para el rastreo de cuadros que sugieren trastornos mentales, especialmente de síntomas ansiosos, depresivos y somáticos, se destacan la Escala de Estrés Psicológico de Kessler (K10) ^(^ 14 ^-^ 16 ^)^ y el *Self Reporting Questionnaire* (SRQ-20) ^(^ 17 ^-^ 18 ^)^ . Estos instrumentos son preferibles debido a la facilidad de aplicación y a las propiedades psicométricas adecuadas evidenciadas en investigaciones anteriores ^(^ 14 ^-^ 16 ^,^ 18 ^-^ 31 ^)^ . 

 Este estudio se centra en la investigación de las propiedades psicométricas de la versión brasileña de la K10 en el contexto de la educación superior. Se trata de una escala de rastreo de sufrimiento psicológico compuesta por diez ítems, los cuales representan síntomas de ansiedad y depresión ^(^ 14 ^-^ 16 ^)^ . Su versión original en inglés demostró una estructura unifactorial con alta consistencia interna (α=0,93) ^(^ 14 ^)^ , además de validez predictiva para el rastreo de sufrimiento psicológico inespecífico ^(^ 15 ^)^ . 

 Varios estudios internacionales confirmaron la fiabilidad de la K10 en distintas poblaciones y culturas, incluyendo adultos portugueses (α=0,91) ^(^ 22 ^)^ , personas mayores (α=0,84) ^(^ 20 ^)^ y adultos brasileños (α=0,87; α=0,93) ^(^ 19 ^,^ 21 ^)^ , adolescentes indonesios (α=0,89) ^(^ 23 ^)^ , trabajadores sociales palestinos (α=0,88) ^(^ 24 ^)^ , usuarios de la atención primaria de salud en Irán (α=0,87) ^(^ 25 ^)^ , pacientes de ambulatorios de medicina general y cuidadores en Uganda (α=0,86) ^(^ 26 ^)^ y en Sudáfrica (α=0,84; ω=0,88) ^(^ 27 ^)^ , pueblos aborígenes (α=0,88; α=0,87; α=0,84) ^(^ 28 ^)^ , muestras clínicas australianas (α=0,93) ^(^ 14 ^,^ 29 ^)^ , además de adultos holandeses, turcos, marroquíes (α=0,93) ^(^ 30 ^)^ y estadounidenses (α=0,89; α=0,93) ^(^ 31 ^)^ . En lo referente a la estructura factorial de la K10, investigaciones previas identificaron modelos unifactoriales ^(^ 15 ^,^ 21 ^,^ 26 ^,^ 28 ^-^ 30 ^)^ , dos factores correlacionados ^(^ 29 ^,^ 31 ^)^ y bifactorial ^(^ 19 ^)^ con dos factores correlacionados, en diversos contextos y poblaciones. 

 La K10 posee traducción en varios idiomas, incluido el portugués brasileño ^(^ 32 ^)^ . No obstante, en Brasil, solo tres estudios ^(^ 19 ^-^ 21 ^)^ se centraron en las evidencias de validez, enfocándose en muestras de personas mayores ^(^ 20 ^)^ y adultos ^(^ 19 ^,^ 21 ^)^ , dejando un vacío en cuanto a su aplicabilidad entre estudiantes universitarios. Así, se destaca la escasez de datos sobre la validez de la versión brasileña de la K10 ^(^ 32 ^)^ , especialmente en relación con su capacidad para rastrear sufrimiento psicológico entre jóvenes y en el contexto de la educación superior. Estudios de esta naturaleza son importantes porque demuestran si el instrumento es capaz de medir adecuadamente el constructo que se propone evaluar, en un determinado contexto y para un grupo de personas. Instrumentos que presentan estructura interna inadecuada conducen a conclusiones equivocadas. Por lo tanto, es fundamental investigar la validez de la estructura interna de una escala antes de utilizarla en investigaciones o evaluaciones ^(^ 33 ^)^ . 

 Conforme a los *Standards for Educational And Psychological Testing* ^(^ 34 ^)^ , la validez no es una propiedad de una escala, sino de los puntajes del instrumento, de tal manera que es necesaria una continua fuente de evidencias de validez para considerar un instrumento válido en un determinado contexto. En este sentido, se entiende que son necesarios nuevos estudios para confirmar y/o añadir nuevas evidencias de validez de la K10 al contexto brasileño. Así, el objetivo de este estudio fue evaluar la validez basada en la estructura interna, validez concurrente y predictiva de la versión brasileña de la Escala de Sufrimiento Psicológico de Kessler, para el rastreo de sufrimiento psicológico en estudiantes de nivel superior. 

## Método

### Tipo del estudio

Se trata de un estudio metodológico, con enfoque cuantitativo.

### Lugar y período de recolección de datos

Los datos fueron recolectados en el período de abril a diciembre de 2019, en dos Instituciones Federales de Educación Superior (IFES) ubicadas en la región noreste de Brasil, con alta vulnerabilidad económica y social. Una de estas instituciones se encuentra en una región del interior.

### Población, muestra y criterios de elegibilidad

 La población estuvo compuesta por estudiantes de nivel superior matriculados en los cursos presenciales de las IFES sedes de la investigación. La estimación muestral fue calculada con la ayuda de una calculadora en línea ^(^ 35 ^)^ , considerando el uso de modelado de ecuaciones estructurales, mediante los siguientes parámetros ^(^ 36 ^)^ : tamaño del efecto a priori: 0,10; poder de la prueba: 0,80; número de variables latentes: 2; número de variables observadas: 10; y nivel de probabilidad: 0,05. Así, la estimación inicial fue de 947 participantes. 

 Para la selección de la muestra, se adoptó la técnica de muestreo por conglomerados, con sorteo simple de las aulas con ayuda del *software Excel* y el comando *Random* , basado en una lista proporcionada por el departamento de matrículas de la institución, que contenía el código de cada grupo y el respectivo curso. Tras el sorteo de las aulas, la captación de los estudiantes se realizó a partir de los siguientes criterios de inclusión: tener edad igual o superior a 18 años y estar en el aula en el horario previamente acordado con el profesor para la recolección de datos. 

Se excluyeron de la investigación los estudiantes que reportaron tener problemas clínicos, como dolores o alguna situación de crisis en salud mental, que pudieran impedir su participación. Este criterio de exclusión se evaluó mediante el relato del participante, inmediatamente después de la explicación de los objetivos de la investigación y de las instrucciones sobre cómo completar los instrumentos. Si el estudiante reportaba cualquier condición clínica que impidiera su participación, el equipo atendía la demanda y proporcionaba la derivación al servicio de psicología de las instituciones.

En cuanto a los criterios de exclusión, también se excluyeron del análisis los instrumentos que presentaron ausencia de datos o tachaduras en la variable edad, aquellos completados solo con datos de caracterización sociodemográfica, y los que tuvieron respuesta afirmativa a la pregunta distractora. En este estudio, la pregunta distractora se utilizó para minimizar el sesgo de respuesta y estaba relacionada con el uso de una sustancia psicoactiva ficticia. Tras la aplicación de estos criterios de elegibilidad, la muestra final de este estudio consistió en 1.034 estudiantes, aunque la estimación inicial preveía 947 participantes.

### Procedimientos de recolección de datos

Inicialmente, se realizó la capacitación del equipo de salud sobre la temática y los instrumentos de recolección de datos. Este equipo incluyó docentes de los cursos de Enfermería y Trabajo Social, estudiantes de grado en Trabajo Social y en Enfermería, y otros profesionales de nivel superior (psicóloga, trabajadora social, enfermero y pedagogo).

A continuación, se contactó a los coordinadores de los cursos para proporcionar explicaciones sobre la investigación, promover la sensibilización de los profesores para la liberación de sus clases y programar la recolección de datos. Después de este contacto, se realizó el sorteo de las aulas que participarían en el estudio, utilizando un método de selección aleatoria simple, basado en una lista que contenía la numeración de las aulas, la asignatura, el turno de la clase y el curso.

Los potenciales participantes fueron invitados a la investigación, siendo informados sobre la naturaleza voluntaria de su participación, los objetivos del estudio, los posibles riesgos y beneficios, además de recibir instrucciones detalladas sobre los instrumentos, el tiempo medio necesario para el llenado (60 minutos) y la importancia del llenado individual. La recolección de datos se llevó a cabo en el aula, en días y horarios previamente acordados con el profesor y el coordinador del curso correspondiente.

### Instrumentos de recolección de datos

 El instrumento de recolección de datos estuvo compuesto por variables de caracterización sociodemográfica (edad, género, orientación sexual, religión, estado civil, número de hijos, ingreso familiar, raza/color de piel auto-referida y con quién residía el estudiante), K10 ^(^ 14 ^,^ 32 ^)^ y SRQ-20 ^(^ 17 ^-^ 18 ^)^ . La K10 es autoaplicable, compuesta por diez ítems relacionados con los síntomas ansiosos y depresivos que una persona experimentó en el período más reciente de 30 días. Estos síntomas juntos representan el sufrimiento psicológico ^(^ 14 ^)^ . Cada ítem se puntúa en una escala Likert de cinco puntos (1 – Todos los días; 2 - La mayor parte de los días; 3 – Algunos días; 4 – Pocos días; 5 – Ningún día). Para calcular los puntajes totales, se debe inicialmente invertir la escala de cinco puntos y, posteriormente, efectuar la suma de las respuestas. Los valores posibles varían de 10 a 50 ^(^ 14 ^)^ . La versión original de la escala, en inglés, reveló alta consistencia interna (α=0,93) ^(^ 14 ^)^ . En cuanto a los puntos de corte, un puntaje mayor que 17 presentó sensibilidad del 81,1% y especificidad del 83,0% para identificar sufrimiento psicológico ^(^ 37 ^)^ . 

 En este estudio, se investigaron tres dimensiones de validez de la versión en portugués brasileño de la K10: validez basada en la estructura interna, validez concurrente y validez predictiva. La validez basada en la estructura interna evalúa en qué medida las correlaciones entre los ítems reflejan la estructura del constructo que el instrumento se propone medir, siendo comúnmente explorada mediante análisis factoriales ^(^ 34 ^)^ . 

 La validez concurrente busca determinar la asociación entre la K10 y otros instrumentos que miden el mismo constructo, mientras que la validez predictiva se enfoca en averiguar si los puntajes de la K10 pueden predecir el desempeño de los participantes en futuras pruebas o comportamientos, específicamente la ocurrencia de sufrimiento psicológico ^(^ 34 ^)^ . Estas valideces fueron examinadas comparando la K10 con el SRQ-20, este último adoptado como patrón oro. 

 El SRQ-20 es autoadministrable, compuesto por 20 ítems relacionados con síntomas no psicóticos (somáticos, depresivos y ansiosos), cuyas respuestas son dicotómicas (sí/no). La puntuación final se obtiene a partir de la suma de cada respuesta afirmativa, variando de 0 a 20 puntos ^(^ 17 ^-^ 18 ^)^ . Para la detección de cuadros sugerentes de TMC en la versión brasileña, se estableció un punto de corte superior a 7,0 puntos, con evidencias de validez de criterio robustas (sensibilidad=86,33% y especificidad=89,31%). Adicionalmente, esta versión específica demostró alta confiabilidad, según el coeficiente alfa de Cronbach (α=0,86) ^(^ 18 ^)^ . 

### Análisis de los datos

 Los datos se organizaron en una hoja de cálculo del *software Excel* y se analizaron con la ayuda de los *softwares* JASP - versión 0.17.2.1 y *MedCalc* - prueba gratuita. En cuanto a la caracterización de la muestra, se presentaron frecuencias y medidas de tendencia central. Para la verificación de las evidencias de validez basadas en la estructura interna de la K10, se utilizó la Modelización por Ecuaciones Estructurales (MEE), aplicando el Análisis Factorial Confirmatorio Bifactor (AFC Bifactor), para comparar los modelos unifactorial ^(^ 15 ^,^ 21 ^,^ 26 ^,^ 28 ^-^ 30 ^)^ , dos factores correlacionados ^(^ 29 ^,^ 31 ^)^ y bifactorial ^(^ 19 ^)^ de la K10 evidenciados en estudios anteriores. 

 Modelos bifactoriales y jerárquicos relacionados han sido incorporados para la conceptualización, estudio y diagnóstico de psicopatologías, y permiten comprender si una variable observada se explica mejor por una dimensión general o por una dimensión específica, a partir de la comparación de varios modelos ^(^ 38 ^-^ 41 ^)^ . 

 En el AFC Bifactor, se utilizó la ortogonalización de las variables latentes, de modo que la varianza explicada de cada variable latente en relación a cada variable observada no se solapara respecto a otras variables latentes del modelo ^(^ 38 ^-^ 39 ^)^ . Además, se aplicó el método de estimación *Robust Diagonally Weighted Least Squares* (RDWLS), adecuado para datos categóricos ^(^ 42 ^-^ 43 ^)^ . La adecuación del modelo se evaluó mediante los índices de ajuste: χ² (chi cuadrado), *Comparative Fit Index* (CFI), *Tucker-Lewis Index* (TLI), *Standardized Root Mean Square Residual* (SRMR) y *Root Mean Square Error of Approximation* (RMSEA). La relación entre el valor de χ² y los grados de libertad (gl) del modelo debe ser menor que 3,0 para indicar un buen ajuste del modelo, mientras que valores por encima de 5,0 indican un ajuste inadecuado. Los valores de CFI y TLI deben ser mayores o iguales a 0,90, siendo preferibles valores por encima de 0,95. El SRMR debe ser máximo 0,08, y el valor de RMSEA debe ser menor o igual a 0,06 o, como máximo, 0,08, con intervalo de confianza (límite superior) menor o igual a 0,10 ^(^ 44 ^)^ . 

 Además de los índices de ajuste mencionados previamente, se procedió a la evaluación de las cargas factoriales estandarizadas en los tres modelos probados, resaltando la importancia de aquellas con valores superiores a 0,40. Estos valores son considerados relevantes ya que indican una contribución significativa de los ítems al factor en cuestión. Junto con este análisis, se examinó la Varianza Media Extraída (VME) para cada factor. La VME cuantifica la porción de la varianza captada por los ítems de un constructo en comparación con la varianza atribuida a errores de medición o a varianzas únicas. Una VME elevada señala que una parte considerable de la varianza de los ítems se comparte con el constructo subyacente, reforzando la consistencia interna del factor. Por el contrario, una VME baja puede sugerir que los ítems del constructo fallan en capturar adecuadamente la varianza común o indicar la presencia de una cantidad significativa de varianza debido a errores de medición. Valores de VME superiores a 0,5 se consideran generalmente como satisfactorios, mientras que aquellos por debajo de este umbral pueden revelar la necesidad de revisión o mejora del modelo ^(^ 45 ^)^ . 

 Para la verificación de la fiabilidad de la estructura factorial, se utilizó el Omega Jerárquico (ωH), indicado para evaluar el grado en que una medida compuesta puede ser explicada por un factor general ^(^ 46 ^)^ . Un valor superior a 0,70 indica una fiabilidad aceptable del puntaje generado por ese factor general ^(^ 39 ^)^ . El modelo cuyos ítems presentaron mayor carga factorial estandarizada, mayor VME, mejores índices de ajuste y de fiabilidad ^(^ 47 ^)^ fue considerado el más apropiado para representar la estructura factorial de la K10. 

 Para verificar la validez concurrente de la K10, considerando el SRQ-20 como patrón oro, se utilizó el coeficiente de correlación r de Pearson, con sus respectivos intervalos de confianza a partir de re-muestreos de *Bootstrapping* , con el fin de corregir problemas de normalidad de los datos. Valores de correlación mayores o iguales a 0,70 sugieren una correlación adecuada ^(^ 34 ^)^ . 

 Finalmente, la validez predictiva fue investigada mediante el análisis de la Curva ROC ( *Receiver Operator Characteristic* ), medidas de precisión (sensibilidad y especificidad) y los respectivos Intervalos de Confianza (IC) de 95% según Clopper-Pearson. El Área Bajo la Curva ROC ( *Area Under The Curve* – AUC) con valor mayor o igual a 0,80, alta sensibilidad y especificidad, indica una precisión adecuada del test para el rastreo de determinado fenómeno, considerando un determinado punto de corte ^(^ 48 ^)^ . 

### Aspectos éticos

La investigación fue aprobada por el Comité de Ética en Investigación con Seres Humanos, bajo el número de dictamen 2.937.477.

## Resultados

La edad varió de 18 a 25 años (media=23,5; desviación estándar=7,17), con mayores proporciones de estudiantes en las franjas de edad de hasta 20 años (n=537; 44,9%) y de 21 a 30 años (n=511; 42,7%). Se observó una proporción similar de hombres (n=596; 49,8%) y mujeres cisgénero (n=581; 48,5%), y el 85,8% (n=1.027) se identificaron como heterosexuales. Un porcentaje del 68,6% (n=821) refirió seguir alguna religión. La mayoría reportó estado civil soltero (n=1.034; 86,4%) y no tener hijos (n=1.069; 89,3%). La renta familiar media fue de R$ 2.847,40. Más de la mitad se autodeclaró de color pardo (n=626; 52,3%) y el 47,0% (n=563) vivía con su madre y su padre.

 En relación a los resultados provenientes de la comparación entre modelos de AFC ( Tabla 1 ), los modelos de dos factores correlacionados y el bifactorial se destacaron por presentar mejores índices de ajuste. Notablemente, el modelo bifactorial exhibió valores superiores en los índices CFI (1,000), TLI (0,999), SRMR (0,0019) y RMSEA (0,028; IC: 0,015 – 0,041). 

**Tabla 1:** - Índices de ajuste de diferentes modelos factoriales de la versión en portugués brasileño de la Escala de Estrés Psicológico de Kessler en una muestra de estudiantes de educación superior (n = 1.197). Recife, PE, Brasil, 2019

**Modelos**	χ ^2^ *	**gl** ^†^	**CFI** ^‡^	**TLI** ^§^	**SRMR** ^||^	**RMSEA** ^¶^	**IC 90%** **	
1 factor	483,697 ^††^	35	0,992	0,989	0,058	0,111	0,103	0,120
2 factores correlacionados	166,066 ^††^	34	0,998	0,997	0,036	0,061	0,052	0,071
Bi-factor	45.895 ^‡‡^	25	1,000	0,999	0,019	0,028	0,015	0,041

*χ ^2^ Chi-square

^†^ gl Grados de libertad

^‡^ CFI *Comparative Fit Index*

^§^ TLI *Tucker-Lewis Index*

^||^ SRMR *Standardized Root Mean Squared Residual*

**^¶^ RMSEA :** *Root Mean Square Error of Approximation*

**IC 90% Intervalo de Confianza de 90%

^††^ p<0,001

^‡‡^ p<0,05

 Sin embargo, es necesario evaluar otras medidas para determinar la estructura factorial más adecuada, incluyendo las cargas factoriales, VME y ωH, especialmente en cuanto al modelo bifactorial, que tiende a presentar *overfitting* , es decir, índices de ajuste sobreestimados. En relación a las cargas factoriales ( Tabla 2 ), aquellas asociadas al factor general - sufrimiento psicológico variaron entre 0,69 y 0,91. Para los factores específicos, las variaciones fueron de 0,22 a 0,90 en el factor - síntomas depresivos y de -0,08 a 0,89 en el factor - síntomas ansiosos. Ítems como 1, 2, 3 y 6 presentaron cargas factoriales inferiores a 0,30 en los factores específicos, sugiriendo una relación variable con los factores e indicando que algunos ítems tienen cargas negativas, especialmente en el factor - síntomas ansiosos. Estos resultados sugieren que la estructura más adecuada para el instrumento incluye un factor general - sufrimiento psicológico, dadas las mayores cargas factoriales observadas en los modelos unifactorial y bifactorial. 

 El análisis de la varianza explicada ( Tabla 2 ) muestra que el modelo unifactorial fue capaz de explicar el 53,8% de la varianza de los ítems, lo que representa un 3,8% más que el factor general - sufrimiento psicológico en el modelo bifactorial (50,0%). En contraste, el modelo de dos factores correlacionados reveló una varianza explicada significativamente mayor en los dos factores (síntomas depresivos – 50,9%; síntomas ansiosos – 73,5%) en comparación con los factores específicos del modelo bifactorial (síntomas depresivos – 20,0%; síntomas ansiosos – 5,0%). 

Esto demuestra que el modelo bifactorial explicó el 75,0% de la varianza total de los ítems, indicando un incremento importante en relación a los otros modelos, con la VME del factor general significativamente superior a la de los factores específicos. Por lo tanto, al controlar el efecto de los factores específicos y sin considerar el número de ítems, el factor general explicó la mayor parte de la varianza de los ítems. En otras palabras, cuando se ajusta por el efecto del factor general, las consistencias internas de los factores específicos se presentan bajas. Esto indica que la variabilidad de los ítems es explicada principalmente por el factor general, reduciendo la relevancia de los factores específicos en la explicación de la varianza de los ítems.

 En cuanto a la fiabilidad de las estructuras factoriales ( Tabla 2 ), el modelo unifactorial y el factor general del modelo bifactorial presentaron los mayores índices de fiabilidad, con omega jerárquico de 0,996 y 0,886, respectivamente. Los modelos de dos factores correlacionados y el modelo bifactorial al considerar los factores específicos, exhibieron índices de fiabilidad más bajos, indicando una menor precisión de estos modelos. En particular, el modelo bifactorial mostró las menores consistencias internas en los factores específicos, cuando se controla el efecto del factor general. 

**Tabla 2:** - Cargas factoriales estandarizadas para los modelos factoriales de la Escala de Estrés Psicológico de Kessler en una muestra de estudiantes de educación superior (n = 1.197). Recife, PE, Brasil, 2019

**Ítem** s	**Carga factorial estandarizada**					
	**Análisis Factorial Confirmatorio tradicional**			**Análisis Factorial Confirmatorio bifactorial**		
	**Modelo unifatorial**	**Modelo de dos factores correlacionados**		**Factor general**	**Factores específicos**	
	SP*	SD ^†^	SA ^‡^	SP*	SD ^†^	SA ^‡^
Ítem 1	0,73	0,75		0,70	0,22	
Ítem 2	0,80		0,86	0,86		-0,08
Ítem 3	0,84		0,89	0,91		-0,23
Ítem 4	0,82	0,84		0,70	0,47	
Ítem 5	0,78		0,83	0,83		0,45
Ítem 6	0,80		0,85	0,83		0,21
Ítem 7	0,89	0,90		0,73	0,56	
Ítem 8	0,78	0,80		0,71	0,35	
Ítem 9	0,88	0,90		0,74	0,52	
Ítem 10	0,84	0,85		0,69	0,53	
Varianza Media Explicada (%)	53,8	50,9	73,5	50,0	20,0	5,0
Omega jerárquico	0,996	0,648	0,460	0,886	0,106	0,016

*SP Sufrimiento psicológico

^†^ SD Síntomas depresivos

^‡^ SA Síntomas ansiosos

Los resultados obtenidos por el AFC revelaron que el modelo bifactorial demostró superioridad en la representación de la estructura de la K10 para esta muestra, evidenciada por diversos aspectos. Primeramente, los índices de ajuste del modelo bifactorial, compuesto por un factor general – sufrimiento psicológico – y dos factores específicos – síntomas depresivos y síntomas ansiosos, fueron significativamente superiores. Además, las cargas factoriales fueron mayores para el factor general, resaltando su preeminencia en la estructura del instrumento e indicando una consistencia interna elevada. Este modelo se distingue también por su capacidad de explicar una mayor porción de la varianza de los ítems.

Comparando el modelo bifactorial con el modelo unifactorial, se percibe que, aunque el último presenta cargas factoriales superiores y una consistencia interna satisfactoria, sus índices de ajuste son inadecuados, lo que confiere al modelo bifactorial una ventaja en este aspecto. El análisis de la varianza explicada por ambos modelos, al evaluar el factor general, resultó similar. Sin embargo, la VME del factor general, junto con los factores específicos en el modelo bifactorial, evidencia una varianza explicada superior a la del modelo unifactorial. Esta comparación refuerza la superioridad del modelo bifactorial, particularmente por su habilidad para capturar una dimensión más amplia de la varianza, fortaleciendo su aplicabilidad y precisión en el análisis de la estructura de la K10 para la muestra estudiada.

 Sobre la validez concurrente, se observó una fuerte correlación entre los puntajes de la K10 y del SRQ-20 de r=0,813 (IC 95% *Bootstrapping* : 0,784 – 0,837; r2=0,660; p<0,001), lo que indica validez concurrente de la K10 para el rastreo de sufrimiento psicológico. En términos de validez predictiva, el mejor punto de corte de la K10 para ese rastreo fue un puntaje total mayor que 21, con una sensibilidad de 85,2% (IC 95%: 81,6 - 88,3) y especificidad de 82,9% (IC 95%: 79,9 - 85,6), ambas elevadas, además de un excelente valor de AUC de 0,915 (IC 95% *Bootstrapping* : 0,896 - 0,929; p<0,0001) ( Figura 1 ). 

**Figura 1 -:** Área bajo la Curva ROC de la Escala de Estrés Psicológico de Kessler, versión en portugués brasileño

*K10 = Escala de Estrés Psicológico de Kessler; ^†^ AUC = *Area Under the Curve* ; ^‡^ P = Valor de p

 Por lo tanto, según estos resultados y utilizando este punto de corte, sería posible detectar correctamente el 85,2% de los casos de sufrimiento psicológico, con una tasa de falsos positivos del 17,1%. Se pueden consultar más detalles en la Tabla 3 . 

**Tabla 3:** - Puntos de corte de la Escala de Estrés Psicológico de Kessler para la detección de sufrimiento psicológico en estudiantes (n = 1.197). Recife, PE, Brasil, 2019

**Punto de corte**	**Sensibilidad**	**IC95%** *	**Especificidad**	**IC95%** *
≥10	100,00	99,2 - 100,0	0,00	0,0 - 0,5
>10	99,79	98,8 - 100,0	10,72	8,6 - 13,2
>11	99,58	98,5 - 99,9	18,66	15,9 - 21,7
>12	99,58	98,5 - 99,9	27,86	24,6 - 31,3
>13	98,94	97,5 - 99,7	35,79	32,3 - 39,4
>14	97,88	96,1 - 99,0	44,01	40,3 - 47,7
>15	97,03	95,1 - 98,4	52,23	48,5 - 55,9
>16	95,76	93,5 - 97,4	59,33	55,6 - 62,9
>17	94,07	91,5 - 96,0	65,18	61,6 - 68,7
>18	92,16	89,4 - 94,4	69,64	66,1 - 73,0
>19	90,68	87,7 - 93,1	74,93	71,6 - 78,1
>20	88,14	84,9 - 90,9	79,39	76,2 - 82,3
>21	85,17	81,6 - 88,3	82,87	79,9 - 85,6
>22	81,36	77,5 - 84,8	84,96	82,1 - 87,5
>23	77,97	74,0 - 81,6	87,74	85,1 - 90,1
>24	73,52	69,3 - 77,4	90,39	88,0 - 92,4
>25	70,76	66,4 - 74,8	91,92	89,7 - 93,8
>26	66,31	61,9 - 70,6	93,45	91,4 - 95,2
>27	61,86	57,3 - 66,3	95,40	93,6 - 96,8
>28	54,66	50,0 - 59,2	96,24	94,6 - 97,5
>29	48,94	44,3 - 53,6	96,80	95,2 - 98,0
>30	44,70	40,2 - 49,3	98,05	96,8 - 98,9
>31	40,04	35,6 - 44,6	98,47	97,3 - 99,2
>32	35,38	31,1 - 39,9	98,89	97,8 - 99,5
>33	33,05	28,8 - 37,5	99,03	98,0 - 99,6
>34	28,81	24,8 - 33,1	99,58	98,8 - 99,9
>35	23,94	20,2 - 28,1	99,72	99,0 - 100,0
>36	20,97	17,4 - 24,9	99,72	99,0 - 100,0
>37	17,80	14,5 - 21,6	99,72	99,0 - 100,0
>38	12,92	10,0 - 16,3	99,72	99,0 - 100,0
>39	10,59	8,0 - 13,7	99,72	99,0 - 100,0
>40	7,84	5,6 - 10,6	99,72	99,0 - 100,0
>41	6,14	4,2 - 8,7	99,86	99,2 - 100,0
>42	5,08	3,3 - 7,5	99,86	99,2 - 100,0
>43	4,45	2,8 - 6,7	99,86	99,2 - 100,0
>44	2,75	1,5 - 4,7	99,86	99,2 - 100,0
>45	2,33	1,2 - 4,1	99,86	99,2 - 100,0
>46	2,12	1,0 - 3,9	100,00	99,5 - 100,0
>47	1,27	0,5 - 2,7	100,00	99,5 - 100,0
>48	0,85	0,2 - 2,2	100,00	99,5 - 100,0
>49	0,64	0,1 - 1,8	100,00	99,5 - 100,0
>50	0,00	0,0 - 0,8	100,00	99,5 - 100,0

*IC95% Intervalo de Confianza del 95% de Clopper-Pearson

## Discusión

 Esta investigación examinó las evidencias de validez de la K10, versión en portugués brasileño, para detectar el sufrimiento psicológico en estudiantes universitarios, basándose en la estructura interna, validez concurrente y validez predictiva. En este estudio, los resultados derivados del modelo de AFC bifactorial mostraron mejores índices de ajuste, en línea con un estudio previo realizado con una muestra de adultos brasileños ^(^ 19 ^)^ . Además, el factor general de sufrimiento psicológico demostró una alta consistencia interna y explicó un porcentaje relevante de la varianza, en consonancia con investigaciones anteriores que señalaron una estructura unifactorial compuesta por el sufrimiento psicológico ^(^ 15 ^,^ 19 ^,^ 26 ^,^ 28 ^-^ 30 ^)^ . 

 Los modelos bifactoriales son especialmente útiles cuando se busca comprender no solo una dimensión global, sino también variaciones específicas en torno a esa dimensión, ofreciendo una visión más profunda y diversa de las relaciones entre las variables que otros modelos de Análisis Factorial ^(^ 38 ^-^ 41 ^)^ . Esta aproximación multifacética es esencial para profundizar en la comprensión de la estructura del sufrimiento psicológico y sus diversas manifestaciones. 

 Desde esta perspectiva, la selección y aplicación de la K10, como han destacado algunos autores ^(^ 19 ^,^ 29 ^)^ , deben estar estrechamente relacionadas con el propósito específico del instrumento y el perfil de los individuos evaluados. En contextos de poblaciones comunitarias y procesos de cribado, la detección del sufrimiento psíquico general, en lugar de cuadros específicos como ansiedad y depresión, resulta más conveniente. Por otro lado, en lo que respecta a la evaluación de una población clínica, la diferenciación entre problemas específicos como ansiedad y depresión parece más apropiada ^(^ 29 ^)^ , lo que indica la flexibilidad de la K10 para satisfacer diferentes necesidades de evaluación. 

 La relevancia de este enfoque es especialmente significativa al considerar a los estudiantes universitarios, entre quienes es notable la presencia concomitante de síntomas ansiosos y depresivos ^(^ 3 ^,^ 10 ^,^ 49 ^-^ 53 ^)^ . Esta realidad respalda la estructura de un factor general de sufrimiento psicológico identificado en este estudio. De hecho, aunque este sufrimiento a menudo se concibe, desde una perspectiva bidimensional, como la coexistencia de síntomas ansiosos y depresivos, tales cuadros actúan como factores de riesgo bidireccionales entre sí ^(^ 54 ^-^ 55 ^)^ . Mientras que la ansiedad se asocia con el miedo, nerviosismo, preocupación excesiva y sensación de tensión, la depresión está relacionada con la tristeza, desesperanza, falta de interés o placer en actividades diarias, fatiga y comportamiento suicida (ya sea por ideación suicida, intento o realización del suicidio) ^(^ 52 ^-^ 53 ^,^ 56 ^)^ . 

 Los autores han evidenciado una alta tasa de comorbilidad entre depresión y ansiedad, con impactos significativos en la funcionalidad de los estudiantes universitarios ^(^ 57 ^-^ 58 ^)^ . La superposición de estos cuadros y sus factores de riesgo indica una estrecha relación entre estos trastornos, sugiriendo que se consideren como manifestaciones de un síndrome psiquiátrico internalizante, caracterizado por síntomas dirigidos hacia el interior, como los síntomas ansiosos y depresivos. Esta concepción se ha propuesto como beneficiosa para el cribado y el tratamiento ^(^ 59 ^)^ . La presencia simultánea de estos cuadros intensifica el sufrimiento psicológico y repercute negativamente en la calidad de vida de los estudiantes ^(^ 57 ^-^ 58 ^)^ . Por lo tanto, es crucial comprender este fenómeno en su totalidad y diversidad de síntomas, con el fin de facilitar el desarrollo e implementación de intervenciones terapéuticas más efectivas. 

 En cuanto a la validez concurrente, la K10 mostró una alta correlación con el SRQ-20, uno de los instrumentos más utilizados para el cribado de trastornos mentales comunes (TMC). Este desempeño de la K10, en términos de validez concurrente, está en línea con estudios anteriores que confirmaron esta propiedad psicométrica ^(^ 14 ^,^ 60 ^)^ . En cuanto al constructo evaluado, ambos instrumentos exploran fenómenos similares, pero con sus especificidades. La K10, compuesta por diez ítems, aborda el sufrimiento psicológico a través de síntomas depresivos y ansiosos, utilizando una escala Likert de cinco puntos para las respuestas ^(^ 14 ^)^ . Por otro lado, el SRQ-20, con sus 20 ítems, investiga síntomas somáticos, depresivos y ansiosos asociados a los TMC, mediante respuestas dicotómicas del tipo sí/no ^(^ 17 ^-^ 18 ^)^ . 

 En cuanto al continuo de respuestas, algunos estudios ^(^ 61 ^-^ 62 ^)^ sugieren que la confiabilidad de las puntuaciones tiende a aumentar a medida que se amplía el número de opciones de respuesta. Sin embargo, otros investigadores ^(^ 63 ^)^ no observaron mejoras significativas en la precisión psicométrica de instrumentos que ofrecen más de seis alternativas de respuesta. Estos señalan que las escalas dicotómicas presentan limitaciones en cuanto a la consistencia interna en comparación con los formatos que ofrecen una gama más amplia de elecciones. Por lo tanto, al seleccionar un instrumento de evaluación, es recomendable considerar no solo la diversidad de opciones de respuesta, sino también la facilidad de aplicación y las propiedades psicométricas validadas en diferentes muestras y estudios previos ^(^ 63 ^)^ . 

 A pesar de ofrecer solo dos opciones de respuesta, el SRQ-20 es ampliamente reconocido y recomendado por la OMS ^(^ 17 ^)^ debido a su simplicidad, bajo costo y buenas propiedades psicométricas. La K10, también caracterizada por su facilidad de uso y costo accesible ^(^ 14 ^)^ , posee propiedades psicométricas sólidas, que fueron reiteradas por los resultados de este estudio. Por lo tanto, la elección entre el SRQ-20 y la K10, aunque ambos evalúan aspectos del sufrimiento psicológico, depende fundamentalmente de las especificidades que el investigador pretende explorar. Si el enfoque es la detección de síntomas somáticos junto con síntomas depresivos y ansiosos, el SRQ-20 resulta más adecuado. Sin embargo, para una investigación más detallada del sufrimiento psicológico desde una perspectiva amplia, que abarque la variedad de síntomas depresivos y ansiosos por medio de una escala Likert, la K10 es la opción más indicada. 

 En esta investigación, la Escala K10 destacó al mostrar un AUC superior, en comparación con los valores encontrados en estudios anteriores, que registraron AUC de 0,90 ^(^ 16 ^)^ , 0,82 ^(^ 23 ^)^ y 0,86 ^(^ 64 ^)^ . Se demostró precisa en la versión en portugués brasileño para el cribado de sufrimiento psicológico entre estudiantes universitarios, alcanzando altas tasas de sensibilidad y especificidad para puntuaciones por encima de 21. La literatura sugiere que los instrumentos de cribado deben exhibir altas tasas de sensibilidad y especificidad en un punto de corte bien definido ^(^ 48 ^)^ . En este estudio, las puntuaciones superiores a 21 cumplieron con estos criterios, en consonancia con los hallazgos de investigaciones que utilizaron la versión en inglés de la K10, donde el punto de corte varió entre 18 y 27 puntos ^(^ 16 ^,^ 23 ^,^ 64 ^)^ . 

Aunque este estudio ofrece evidencias prometedoras sobre la aplicabilidad de la versión en portugués brasileño de la K10 en el contexto de la educación superior, es necesario considerar sus limitaciones, como la realización en solo dos IFES, lo que limita la generalización de los resultados a otras IFES brasileñas. Se sugiere, por lo tanto, un examen más detallado de las propiedades psicométricas de la K10, incluso mediante la aplicación de la Teoría de Respuesta al Ítem (TRI), en muestras de diferentes regiones del país, para permitir el continuo refinamiento de este instrumento.

A pesar de estas limitaciones, queda claro que la estructura global de la K10, representada en este estudio por un factor general de sufrimiento psicológico, presenta validez basada en la estructura interna, validez concurrente y predictiva, y se establece como un instrumento confiable para el cribado del sufrimiento psicológico en estudiantes de nivel superior. Esta herramienta puede contribuir significativamente a la elaboración e implementación de intervenciones en salud que buscan prevenir este cuadro en el contexto académico.

## Conclusión

Este estudio proporciona evidencia sólida sobre la validez de la K10, respaldada por la estructura interna y las validaciones concurrente y predictiva, que ayudarán en la detección del sufrimiento psicológico en estudiantes universitarios. El AFC Bifatorial reveló excelentes índices de ajuste para el modelo bifactorial, en el cual un factor general —sufrimiento psicológico — explicó la mayor parte de la variabilidad de los ítems de la K10 y mostró una alta confiabilidad. Además, la escala demostró validez concurrente y predictiva. Se identificó el punto de corte ideal de la K10 para la detección del sufrimiento psicológico en estos estudiantes, un valor en línea con los resultados observados en investigaciones anteriores con la versión original.

# Translation: Validade da escala de Sofrimento Psicológico de Kessler em estudantes brasileiros do ensino superior *

**DOI:** 10.1590/1518-8345.7073.4255

**Published:** 2024-11-15

**Authors:** Jaqueline Galdino Albuquerque Perrelli, Gabriel Vinícius Souza de Vasconcelos, Jéssica Rodrigues Correia e Sá, Pollyanna Fausta Pimentel de Medeiros, Roberta Uchôa, Zila Sanchez

**Affiliations:** ^1^ Universidade Federal de São Paulo, Departamento de Medicina Preventiva, São Paulo, SP, Brasil.; ^2^ Universidade Federal de Pernambuco, Departamento de Enfermagem, Recife, PE, Brasil.; ^3^ Universidade Federal de Pernambuco, Departamento de Serviço Social, Recife, PE, Brasil.; ^4^ Bolsista do Conselho Nacional de Desenvolvimento Científico e Tecnológico (CNPq), Brasil.; ^5^ Universidade Federal de Pernambuco, Recife, PE, Brasil.; ^6^ Faculdade de Medicina de Olinda, Unidade Curricular Integração Academia, Serviço e Comunidade, Olinda, PE, Brasil.; ^7^ Centro Universitário Maurício de Nassau, Graças, Recife, PE, Brasil.

**Keywords:** Estresse Psicológico, Estudo de Validação, Estudantes, Transtornos Mentais, Educação Superior, Reprodutibilidade dos Testes

## Abstract

avaliar a validade baseada na estrutura interna, validade concorrente e preditiva da versão brasileira da Escala de Sofrimento Psicológico de Kessler, para o rastreamento de sofrimento psicológico em estudantes de nível superior.

estudo metodológico com 1.034 participantes, aplicando-se a Escala de Sofrimento Psicológico de Kessler e o *Self Reporting Questionnaire* . A análise da estrutura interna foi investigada por meio da Análise Fatorial Confirmatória bifatorial, avaliando-se os índices de ajuste e o coeficiente de confiabilidade ômega hierarquizado. A validade concorrente foi verificada pelo teste de correlação de Pearson, enquanto para a validade preditiva utilizou-se sensibilidade, especificidade, área sob a curva ROC e intervalo de confiança de 95%.

o modelo bifatorial apresentou excelentes índices de ajuste (CFI=1,000; TLI=0,999; SRMR=0,019; RMSEA=0,028; IC95%: 0,015 – 0,041) e elevada confiabilidade (ωH=0,886). Houve forte correlação significativa entre a K10 e o SRQ (r=0,813; IC95%: 0,784 – 0,837). O ponto de corte ideal para rastreamento foi identificado como superior a 21, com sensibilidade de 85,2% e especificidade de 82,9%.

a estrutura composta por um fator geral - sofrimento psicológico apresentou elevada confiabilidade. A escala apresentou validade concorrente e preditiva para o rastreio de sofrimento psicológico entre estudantes universitários de ensino superior.

## Destaques

**(1)** Validade baseada na estrutura interna demonstrou estrutura de um fator geral.

**(2)** Elevada confiabilidade da estrutura de um fator geral.

**(3)** Validade convergente da escala tendo o SRQ-20 como padrão-ouro.

**(4)** Validade preditiva para triagem de sofrimento psicológico.

**(5)** Sensibilidade e especificidade elevadas para ponto de corte maior do que 21 pontos.

## Introdução

 A formação acadêmica no ensino superior é marcada por uma série de desafios de naturezas pessoal, social, acadêmica e institucional. Embora a fase propicie experiências enriquecedoras, também é um período em que episódios de sofrimento psicológico podem ser intensificados, especialmente entre os jovens. Tais episódios frequentemente decorrem de fatores como separação familiar, necessidade de estabelecer novas relações sociais, aumento da autonomia e da responsabilidade, elevados níveis de estresse, problemas de sono, pressão por competitividade, dificuldades no desenvolvimento de habilidades e competências essenciais à vida acadêmica ^(^ 1 ^-^ 3 ^)^ . 

 Estima-se que cerca de 15% dos adultos em idade produtiva, incluindo estudantes de nível superior, enfrentam ou enfrentarão transtornos mentais ou episódios de sofrimento psicológico em algum momento de suas vidas ^(^ 4 ^)^ . Entre eles, os Transtornos Mentais Comuns (TMC), como a ansiedade e depressão, são particularmente prevalentes. Os sintomas variam de leves a graves e incluem alterações de humor, pensamento, comportamento e saúde física que afetam o funcionamento e a qualidade de vida, além de estarem associados ao maior risco de suicídio ^(^ 5 ^-^ 6 ^)^ . 

 Revisões sistemáticas destacaram a elevada prevalência de ansiedade, depressão, comportamento suicida, transtornos alimentares, estresse pós-traumático e distúrbios do sono entre universitários, não apenas em países desenvolvidos ^(^ 7 ^-^ 8 ^)^ , mas também em países de baixa e média renda ^(^ 9 ^)^ , onde mais de 80% da carga global de depressão e ansiedade está concentrada ^(^ 9 ^)^ . No Brasil, a situação não é diferente, com altas taxas de prevalência para ansiedade (37,7%), depressão (28,5%) e comportamento suicida (9,1%) ^(^ 10 ^)^ . Contudo, observa-se uma lacuna significativa de levantamentos nacionais brasileiros com amostras representativas de estudantes de nível superior, especialmente nas regiões Norte e Nordeste, as quais enfrentam maiores desigualdades sociais ^(^ 10 ^)^ . 

 A promoção da saúde mental e do bem-estar está presente na meta 3.4 dos Objetivos de Desenvolvimento Sustentável (ODS) da Organização das Nações Unidas (ONU), reforçando a necessidade global de enfrentar esses desafios de forma integrada e inclusiva ^(^ 11 ^)^ . Tal contexto reforça a importância de rastrear quadros sugestivos de transtornos mentais em estudantes que ingressam no ensino superior, particularmente naqueles situados em regiões com desigualdades sociais pronunciadas. Essas condições não apenas favorecem o desenvolvimento de transtornos mentais ^(^ 12 ^)^ , mas também sublinham a necessidade de estudos que fundamentem a implementação de programas voltados à prevenção do sofrimento psicológico e à promoção da saúde mental ^(^ 13 ^)^ , sobretudo no contexto da educação superior. 

 No entanto, mais do que estimar a prevalência do fenômeno, faz-se necessário escolher a ferramenta mais adequada para fornecer dados confiáveis sobre o cenário em questão. Nessa perspectiva, entre os instrumentos recomendados pela Organização Mundial da Saúde (OMS) para o rastreio de quadros que sugerem transtornos mentais, especialmente de sintomas ansiosos, depressivos e somáticos, destacam-se a Escala de Estresse Psicológico de Kessler (K10) ^(^ 14 ^-^ 16 ^)^ e o *Self Reporting Questionnaire* (SRQ-20) ^(^ 17 ^-^ 18 ^)^ . Esses instrumentos são preferíveis devido à facilidade de aplicação e às propriedades psicométricas adequadas evidenciadas em pesquisas anteriores ^(^ 14 ^-^ 16 ^,^ 18 ^-^ 31 ^)^ . 

 Diante do exposto, este estudo centra-se na investigação das propriedades psicométricas da versão brasileira da K10 no contexto do ensino superior. Trata-se de uma escala de rastreamento de sofrimento psicológico composta por dez itens, os quais representam sintomas de ansiedade e depressão ^(^ 14 ^-^ 16 ^)^ . Sua versão original em inglês demonstrou uma estrutura unifatorial com elevada consistência interna (α=0,93) ^(^ 14 ^)^ , além de validade preditiva para o rastreamento de sofrimento psicológico inespecífico ^(^ 15 ^)^ . 

 Diversas pesquisas internacionais confirmaram a confiabilidade da K10 em distintas populações e culturas, incluindo adultos portugueses (α=0,91) ^(^ 22 ^)^ , idosos (α=0,84) ^(^ 20 ^)^ e adultos brasileiros (α=0,87; α=0,93) ^(^ 19 ^,^ 21 ^)^ , adolescentes indonésios (α=0,89) ^(^ 23 ^)^ , assistentes sociais palestinos (α=0,88) ^(^ 24 ^)^ , usuários da atenção primária à saúde no Irã (α=0,87) ^(^ 25 ^)^ , pacientes de ambulatórios de medicina geral e cuidadores na Uganda (α=0,86) ^(^ 26 ^)^ , na África do Sul (α=0,84; ω=0,88) ^(^ 27 ^)^ , povos aborígenes (α=0,88; α=0,87; α=0,84) ^(^ 28 ^)^ , amostras clínicas australianas (α=0,93) ^(^ 14 ^,^ 29 ^)^ , além de adultos holandeses, turcos, marroquinos (α=0,93) ^(^ 30 ^)^ e americanos (α=0,89; α=0,93) ^(^ 31 ^)^ . No tocante à estrutura fatorial da K10, investigações prévias identificaram modelos unifatorial ^(^ 15 ^,^ 21 ^,^ 26 ^,^ 28 ^-^ 30 ^)^ , dois fatores correlacionados ^(^ 29 ^,^ 31 ^)^ e bifatorial ^(^ 19 ^)^ com dois fatores correlacionados, em diversos contextos e populações. 

 A K10 possui tradução em vários idiomas, inclusive o português brasileiro ^(^ 32 ^)^ . No entanto, no Brasil, apenas três estudos ^(^ 19 ^-^ 21 ^)^ focaram nas evidências de validade, concentrando-se em amostras de idosos ^(^ 20 ^)^ e adultos ^(^ 19 ^,^ 21 ^)^ , deixando uma lacuna quanto à sua aplicabilidade entre estudantes universitários. Assim, destaca-se a escassez de dados sobre a validade da versão brasileira da K10 ^(^ 32 ^)^ , especialmente em relação à sua capacidade de rastrear sofrimento psicológico entre jovens e no contexto do ensino superior. Estudos dessa natureza são importantes pois demonstram se o instrumento é capaz de medir adequadamente o construto que se propõe a avaliar, em um determinado contexto e para um grupo de pessoas. Instrumentos que apresentam estrutura interna inadequada levam a conclusões equivocadas. Logo, é fundamental que seja investigada a validade da estrutura interna de uma escala antes de utilizá-la em pesquisas ou avaliações ^(^ 33 ^)^ . 

 Conforme o *Standards for Educational And Psychological Testing* ^(^ 34 ^)^ , a validade não é uma propriedade de uma escala, mas sim dos escores do instrumento, de tal modo que é necessária uma contínua fonte de evidências de validade para se considerar um instrumento válido em um determinado contexto. Nesse sentido, entende-se que novos estudos são necessários para confirmar e/ou adicionar novas evidências de validade da K10 ao contexto brasileiro. Assim, o objetivo deste estudo foi avaliar a validade baseada na estrutura interna, a validade concorrente e preditiva da versão brasileira da Escala de Sofrimento Psicológico de Kessler, para o rastreamento de sofrimento psicológico em estudantes de nível superior. 

## Método

### Tipo do estudo

Trata-se de um estudo metodológico, com abordagem quantitativa.

### Local e período de coleta de dados

Os dados foram coletados no período de abril a dezembro de 2019, em duas Instituições Federais de Ensino Superior (IFES) situadas na região Nordeste do Brasil, com elevada vulnerabilidade econômica e social. Uma dessas instituições situa-se em uma região interiorana.

### População, amostra e critérios de elegibilidade

 A população foi composta por estudantes de nível superior matriculados nos cursos presenciais das IFES *loci* da pesquisa. A estimativa amostral foi calculada com o auxílio de uma calculadora *online* ^(^ 35 ^)^ , considerando o uso de Modelagem por Equações Estruturais, por meio dos seguintes parâmetros ^(^ 36 ^)^ : tamanho de efeito *a priori* : 0,10; poder do teste: 0,80; número de variáveis latentes: 2; número de variáveis observadas: 10; e nível de probabilidade: 0,05. Assim, a estimativa inicial foi de 947 participantes. 

 Para a seleção da amostra, adotou-se a técnica de amostragem por *cluster* , com sorteio simples das salas de aula, auxílio do *software* Excel e o comando *Random* , com base em uma lista disponibilizada pelo setor de matrículas da instituição, contendo o código de cada turma e o respectivo curso. Após o sorteio das salas, a captação dos estudantes ocorreu a partir dos seguintes critérios de inclusão: ter idade maior ou igual a 18 anos e estar em sala de aula no horário previamente combinado com o professor para a coleta de dados. 

Excluíram-se da pesquisa os estudantes que relataram ter problemas clínicos, como dores ou alguma situação de crise em saúde mental que pudesse impedir sua participação. Avaliou-se esse critério de exclusão por meio do relato do participante, imediatamente após a explicação dos objetivos da pesquisa e das instruções sobre como preencher os instrumentos. Se o estudante relatasse qualquer condição clínica que impedisse a sua participação, a equipe acolhia a demanda e providenciava o encaminhamento para o serviço de psicologia das instituições.

Ainda quanto aos critérios de exclusão, também foram excluídos da análise os instrumentos que apresentaram ausência de dados ou rasuras na variável idade, aqueles preenchidos apenas com dados de caracterização sociodemográfica, e os que tiveram resposta afirmativa à pergunta distratora. Neste estudo, a pergunta distratora foi utilizada para minimizar o viés de resposta e estava relacionada ao uso de uma substância psicoativa fictícia. Após a aplicação desses critérios de elegibilidade, a amostra final deste estudo consistiu em 1.034 estudantes, embora a estimativa inicial previsse 947 participantes.

### Procedimentos de coleta de dados

Inicialmente, realizou-se o treinamento da equipe de saúde sobre a temática e os instrumentos de coleta de dados. Essa equipe incluiu docentes e estudantes dos cursos de graduação de Enfermagem e de Serviço Social, e outros profissionais de nível superior (psicóloga, assistente social, enfermeiro e pedagogo).

Em seguida, contataram-se os coordenadores dos cursos para fornecer esclarecimentos sobre a pesquisa, promover a sensibilização dos professores para a liberação de suas aulas e agendar a coleta de dados. Após o contato, realizou-se o sorteio das salas de aula que participariam do estudo, utilizando-se de um método de seleção aleatória simples, baseado em uma lista que continha a numeração das salas, a disciplina, o turno da aula e o curso.

Os potenciais participantes foram convidados para a pesquisa, sendo informados sobre a natureza voluntária de sua participação, os objetivos do estudo, os possíveis riscos e benefícios, além de receberem instruções detalhadas sobre os instrumentos, o tempo médio necessário para o preenchimento (60 minutos) e a importância do preenchimento individual. A coleta de dados foi realizada em sala de aula, em dias e horários previamente combinados com o professor e o coordenador do curso correspondente.

### Instrumentos de coleta de dados

 O instrumento de coleta de dados foi composto por variáveis de caracterização sociodemográfica (idade, gênero, orientação sexual, religião, estado civil, número de filhos, renda familiar, raça/cor da pele autorreferida e com quem o estudante residia), K10 ^(^ 14 ^,^ 32 ^)^ e SRQ-20 ^(^ 17 ^-^ 18 ^)^ . A K10 é autoaplicável, composta por dez itens relacionados aos sintomas ansiosos e depressivos que uma pessoa experimentou no período mais recente de 30 dias. Esses sintomas juntos representam o sofrimento psicológico ^(^ 14 ^)^ . Cada item é pontuado em uma escala Likert de cinco pontos (1 – Todos os dias; 2 – A maior parte dos dias; 3 – Alguns dias; 4 – Poucos dias; 5 – Nenhum dia). Para calcular os escores totais, deve-se inicialmente inverter a escala de cinco pontos e, posteriormente, efetuar o somatório das respostas. Os valores possíveis variam de dez a 50 ^(^ 14 ^)^ . A versão original da escala, em inglês, revelou elevada consistência interna (α=0,93) ^(^ 14 ^)^ . Quanto aos pontos de corte, uma pontuação maior do que 17 apresentou sensibilidade de 81,1% e especificidade de 83,0% para identificar sofrimento psicológico ^(^ 37 ^)^ . 

 Neste estudo, foram investigadas três dimensões de validade da versão em português brasileiro da K10: validade baseada na estrutura interna, validade concorrente e validade preditiva. A validade baseada na estrutura interna avalia em que medida as correlações entre os itens refletem a estrutura do construto que o instrumento se propõe a medir, sendo comumente explorada por meio de análises fatoriais ^(^ 34 ^)^ . 

 A validade concorrente busca determinar a associação entre a K10 e outros instrumentos que mensuram o mesmo construto, enquanto a validade preditiva foca em averiguar se os escores da K10 podem prever o desempenho dos participantes em futuros testes ou comportamentos, especificamente a ocorrência de sofrimento psicológico ^(^ 34 ^)^ . Essas validades foram examinadas comparando-se a K10 com o SRQ-20, este último adotado como padrão-ouro. 

 O SRQ-20 é autoaplicável, composto por 20 itens relacionados com sintomas não psicóticos (somáticos, depressivos e ansiosos), cujas respostas são dicotômicas (sim/não). O escore final é obtido a partir do somatório de cada resposta afirmativa, varia de zero a 20 pontos ^(^ 17 ^-^ 18 ^)^ . Para fins de detecção de quadro sugestivo de TMC, na versão brasileira, estabeleceu-se um ponto de corte a superior 7,0 pontos, com evidências de validade de critério robustas (sensibilidade=86,33% e especificidade=89,31%). Adicionalmente, essa versão específica demonstrou elevada confiabildade, conforme o coeficiente alfa de Cronbach (α=0,86) ^(^ 18 ^)^ . 

### Análise dos dados

 Os dados foram organizados em uma planilha do *software* Excel e analisados com o auxílio dos *softwares* JASP - versão 0.17.2.1 e do MedCalc - *free trial* . Quanto à caracterização da amostra, foram apresentadas frequências e medidas de tendência central. Para a verificação das evidências de validade baseadas na estrutura interna da K10, utilizou-se a Modelagem por Equações Estruturais, com a aplicação da Análise Fatorial Confirmatória Bifator (AFC Bifator) para comparar os modelos unifatorial ^(^ 15 ^,^ 21 ^,^ 26 ^,^ 28 ^-^ 30 ^)^ , de dois fatores correlacionados ^(^ 29 ^,^ 31 ^)^ e bifatorial ^(^ 19 ^)^ da K10 evidenciados em estudos anteriores. 

 Modelos bifatoriais e hierárquicos relacionados têm sido incorporados para conceituação, estudo e diagnóstico de psicopatologias e possibilitam compreender se determinada variável observável é melhor explicada por uma dimensão geral ou por uma dimensão específica, a partir de comparação de vários modelos ^(^ 38 ^-^ 41 ^)^ . 

 Na AFC Bifatorial, utilizou-se ortogonalização das variáveis latentes, de modo que a variância explicada de cada variável latente em relação a cada variável observável não se sobrepusesse em relação a outras variáveis latentes do modelo ^(^ 38 ^-^ 39 ^)^ . Ademais, aplicou-se o método de estimação *Robust Diagonally Weighted Least Squares* (RDWLS), que é apropriado para dados categóricos ^(^ 42 ^-^ 43 ^)^ . A adequação do modelo foi avaliada por meio dos índices de ajuste: χ² (qui-quadrado), *Comparative Fit Index* (CFI), *Tucker-Lewis Index* (TLI), *Standardized Root Mean Square Residual* (SRMR) e *Root Mean Square Error of Approximation* (RMSEA). A relação entre o valor de χ² e os graus de liberdade (gl) do modelo deve ser menor que três para indicar um bom ajuste do modelo, enquanto valores acima de cinco indicam um ajuste inadequado. Os valores de CFI e TLI devem ser maiores ou iguais a 0,90, sendo preferíveis valores acima de 0,95. O SRMR deve ser no máximo 0,08, e o valor de RMSEA deve ser menor ou igual a 0,06 ou, no máximo, 0,08, com intervalo de confiança (limite superior) menor ou igual a 0,10 ^(^ 44 ^)^ . 

 Além dos índices de ajuste previamente citados, procedeu-se à avaliação das cargas fatoriais padronizadas nos três modelos testados, destacando-se a importância daquelas com valores superiores a 0,40. Esses valores são considerados relevantes pois indicam uma contribuição significativa dos itens para o fator em questão. Juntamente com essa análise, verificou-se a Variância Média Extraída (VME) para cada fator. A VME quantifica a porção da variância captada pelos itens de um construto em comparação à variância atribuída a erros de medição ou a variâncias únicas. Uma VME elevada sinaliza que uma parte considerável da variância dos itens é compartilhada com o construto subjacente, reforçando a consistência interna do fator. Contrariamente, uma VME baixa pode sugerir que os itens do construto falham em capturar adequadamente a variância comum ou indicar a presença de uma quantidade significativa de variância devida a erros de medição. Valores de VME superiores a 0,5 são geralmente vistos como satisfatórios, enquanto aqueles abaixo deste patamar podem revelar a necessidade de revisão ou aprimoramento do modelo ^(^ 45 ^)^ . 

 Quanto à investigação da fidedignidade da estrutura fatorial, foi utilizado o Ômega Hierárquico (wH), indicado para avaliar o grau em que uma medida composta pode ser explicada por um fator geral ^(^ 46 ^)^ . Um valor superior a 0,70 indica confiabilidade aceitável do escore gerado por esse fator geral ^(^ 39 ^)^ . O modelo cujos itens apresentaram maior carga fatorial padronizada, maior VME, melhores índices de ajuste e de fidedignidade ^(^ 47 ^)^ foi considerado o mais apropriado para representar a estrutura fatorial da K10. 

 Para verificar a validade concorrente da K10, considerando o SRQ-20 como padrão ouro, utilizou-se o coeficiente de correlação r de Pearson, com seus respectivos intervalos de confiança a partir de reamostragens de *Bootstrapping* , com vistas a corrigir problemas de normalidade dos dados. Valores de correlação maiores ou iguais a 0,70 sugerem correlação adequada ^(^ 34 ^)^ . 

 Por fim, a validade preditiva foi investigada por meio da análise da Curva ROC ( *Receiver Operator Characteristic* ), medidas de acurácia (sensibilidade e especificidade) e os respectivos Intervalos de Confiança (IC) de 95% segundo Clopper-Pearson. A Área sob a Curva ROC ( *Area Under The Curve – AUC* ) com valor maior ou igual a 0,80, elevada sensibilidade e especificidade, indica adequada precisão do teste para o rastreamento de determinado fenômeno, considerando um determinado ponto de corte ^(^ 48 ^)^ . 

### Aspectos éticos

A pesquisa foi aprovada pelo Comitê de Ética em Pesquisa com Seres Humanos, sob número do parecer 2.937.477.

## Resultados

A idade variou de 18 a 25 anos (média=23,5; desvio-padrão=7,17), com maiores proporções de estudantes nas faixas etárias de até 20 anos (n=537; 44,9%) e de 21 a 30 anos (n=511; 42,7%). Observou-se uma proporção semelhante de homens (n=596; 49,8%) e mulheres cisgênero (n=581; 48,5%), e 85,8% (n=1.027) se identificaram como heterossexuais. Um percentual de 68,6% (n 821) referiu seguir alguma religião. A maioria relatou estado civil solteiro (n=1.034; 86,4%) e não ter filhos (n=1.069; 89,3%). A renda familiar média foi de R$ 2.847,40. Mais da metade se autodeclarou de cor parda (n=626; 52,3%) e 47,0% (n=563) residiam com mãe e pai.

 Em relação aos resultados provenientes da comparação entre modelos de AFC ( Tabela 1 ), os modelos de dois fatores correlacionados e o bifatorial destacaram-se por apresentarem melhores índices de ajuste. Notadamente, o modelo bifatorial exibiu valores superiores nos índices CFI (1,000), TLI (0,999), SRMR (0,0019) e RMSEA (0,028; IC: 0,015 – 0,041). 

**Tabela 1 -:** Índices de ajuste de diferentes modelos fatoriais da versão em português brasileiro da Escala de Estresse Psicológico de Kessler em uma amostra de estudantes de ensino superior (n = 1.197). Recife, PE, Brasil, 2019

**Modelos**	χ ^2^ *	**gl** ^†^	**CFI** ^‡^	**TLI** ^§^	**SRMR** ^||^	**RMSEA** ^¶^	**IC 90%** **	
1 fator	483,697 ^††^	35	0,992	0,989	0,058	0,111	0,103	0,120
2 fatores correlacionados	166,066 ^††^	34	0,998	0,997	0,036	0,061	0,052	0,071
*Bi-factor*	45.895 ^‡‡^	25	1,000	0,999	0,019	0,028	0,015	0,041

^*^ χ ^2^ Chi-square

^†^ gl Graus de liberdade

^‡^ CFI *Comparative Fit Index*

^§^ TLI *Tucker-Lewis Index*

^||^ SRMR *Standardized Root Mean Squared Residual*

**^¶^ RMSEA :** *Root Mean Square Error of Approximation*

^**^ IC 90% Intervalo de Confiança de 90%

^††^ p<0,001

^‡‡^ p<0,05

 No entanto, é necessário avaliar outras medidas para determinar a estrutura fatorial mais adequada, incluindo cargas fatoriais, VME e wH, especialmente quanto ao modelo bifatorial, que tende a apresentar *overfitting* , ou seja, índices de ajuste superestimados. Em relação às cargas fatoriais ( Tabela 2 ), ou seja, aquelas associadas ao fator geral – sofrimento psicológico variaram entre 0,69 e 0,91. Para os fatores específicos, as variações foram de 0,22 a 0,90 no fator “sintomas depressivos” e de -0,08 a 0,89 no fator “sintomas ansiosos”. Itens como 1, 2, 3 e 6 apresentaram cargas fatoriais inferiores a 0,30 nos fatores específicos, sugerindo uma relação variável com os fatores e indicando que alguns itens têm cargas negativas, especialmente no fator “sintomas ansiosos”. Os resultados sugerem que a estrutura mais adequada para o instrumento inclui um fator geral “sofrimento psicológico”, dadas as maiores cargas fatoriais observadas nos modelos unifatorial e bifatorial. 

 A análise da variância explicada ( Tabela 2 ) mostra que o modelo unifatorial foi capaz de explicar 53,8% da variância dos itens, o que representa 3,8% a mais do que o fator geral “sofrimento psicológico” no modelo bifatorial (50,0%). Em contraste, o modelo de dois fatores correlacionados revelou uma variância explicada significativamente maior nos dois fatores (sintomas depressivos – 50,9%; sintomas ansiosos – 73,5%) em comparação aos fatores específicos do modelo bifatorial (sintomas depressivos – 20,0%; sintomas ansiosos – 5,0%). 

Isso demonstra que o modelo bifatorial explicou 75,0% da variância total dos itens, indicando um incremento importante em relação aos outros modelos, com a VME do fator geral significativamente superior à dos fatores específicos. Logo, ao controlar o efeito dos fatores específicos e desconsiderando o número de itens, o fator geral explicou a maior parte da variância dos itens. Em outras palavras, quando se ajusta para o efeito do fator geral, as consistências internas dos fatores específicos apresentam-se baixas. Isso indica que a variabilidade dos itens é majoritariamente explicada pelo fator geral, reduzindo a relevância dos fatores específicos na explicação da variância dos itens.

 Quanto à confiabilidade das estruturas fatoriais ( Tabela 2 ), o modelo unifatorial e o fator geral do modelo bifatorial apresentaram os maiores índices de confiabilidade, com ômega hierárquico de 0,996 e 0,886, respectivamente. Os modelos de dois fatores correlacionados e o modelo bifatorial ao considerar os fatores específicos, exibiram índices de confiabilidade mais baixos, indicando uma menor precisão desses modelos. Em particular, o modelo bifatorial mostrou as menores consistências internas nos fatores específico, quando controlado o efeito do fator geral. 

**Tabela 2 -:** Cargas fatoriais padronizadas para os modelos fatoriais da Escala de Estresse Psicológico de Kessler em uma amostra de estudantes de ensino superior (n = 1.197). Recife, PE, Brasil, 2019

**Itens**	**Carga fatorial padronizada**					
	**Análise Fatorial Confirmatória tradicional**			**Análise Fatorial Confirmatória bifatorial**		
	**Modelo unifatorial**	**Modelo de dois fatores correlacionados**		**Fator geral**	**Fatores específicos**	
	SP*	SD ^†^	SA ^‡^	SP*	SD ^†^	SA ^‡^
Item 1	0,73	0,75		0,70	0,22	
Item 2	0,80		0,86	0,86		-0,08
Item 3	0,84		0,89	0,91		-0,23
Item 4	0,82	0,84		0,70	0,47	
Item 5	0,78		0,83	0,83		0,45
Item 6	0,80		0,85	0,83		0,21
Item 7	0,89	0,90		0,73	0,56	
Item 8	0,78	0,80		0,71	0,35	
Item 9	0,88	0,90		0,74	0,52	
Item 10	0,84	0,85		0,69	0,53	
Variância Média Explicada (%)	53,8	50,9	73,5	50,0	20,0	5,0
Ômega hierárquico	0,996	0,648	0,460	0,886	0,106	0,016

^*^ SP Sofrimento psicológico

^†^ SD Sintomas depressivos

^‡^ SA Sintomas ansiosos

Os resultados obtidos pela AFC revelaram que o modelo bifatorial demonstrou superioridade na representação da estrutura da K10 para esta amostra, evidenciada por diversos aspectos. Primeiramente, os índices de ajuste do modelo bifatorial, composto por um fator geral – sofrimento psicológico – e dois fatores específicos – sintomas depressivos e sintomas ansiosos, foram significativamente superiores. Além disso, as cargas fatoriais foram maiores para o fator geral, ressaltando sua preponderância na estrutura do instrumento e indicando uma consistência interna elevada. Esse modelo se distingue também pela sua capacidade de explicar uma maior porção da variância dos itens.

Comparando o modelo bifatorial ao modelo unifatorial, percebe-se que, embora o último apresente cargas fatoriais superiores e uma consistência interna satisfatória, seus índices de ajuste são inadequados, o que confere ao modelo bifatorial uma vantagem nesse quesito. A análise da variância explicada por ambos os modelos, ao avaliar o fator geral, mostrou-se similar. Contudo, a VME do fator geral, juntamente com os fatores específicos no modelo bifatorial, evidencia uma variância explicada superior à do modelo unifatorial. A comparação reforça a superioridade do modelo bifatorial, particularmente pela sua habilidade em capturar uma dimensão mais ampla da variância, fortalecendo sua aplicabilidade e precisão na análise da estrutura da K10 para a amostra estudada.

 Sobre a validade concorrente, observou-se forte correlação entre os escores da K10 e do SRQ-20 de r=0,813 (IC 95% *Bootstrapping* : 0,784 – 0,837; r ^2^ =0,660; p<0,001), o que indica validade concorrente da K10 para rastreio de sofrimento psicológico. Em termos de validade preditiva, o melhor ponto de corte da K10 para esse rastreio foi um escore total maior do que 21, com sensibilidade de 85,2% (IC 95%: 81,6 - 88,3) e especificidade de 82,9% (IC 95%: 79,9 - 85,6), ambas elevadas, além de um excelente valor de AUC de 0,915 (IC 95% *Bootstrapping* : 0,896 - 0,929; p<0,0001) ( Figura 1 ). 

**Figura 1 -:** Área sob a Curva ROC da Escala de Estresse Psicológico de Kessler, versão em português brasileiro

*K10 = Escala de Estresse Psicológico de Kessler; ^†^ AUC = *Area Under the Curve* ; ^‡^ P = Valor de p

 Assim, com base nesses resultados e a partir desse ponto de corte, torna-se possível rastrear corretamente 85,2% dos casos de sofrimento psicológico, apresentando uma taxa de falso positivo de 17,1. Outros detalhes podem ser consultados na Tabela 3 . 

**Tabela 3 -:** Pontos de corte da Escala de Estresse Psicológico de Kessler para rastreio de sofrimento psicológico em estudantes (n = 1.197). Recife, PE, Brasil, 2019

**Ponto de corte**	**Sensibilidade**	**IC95%** *	**Especificidade**	**IC95%** *
≥10	100,00	99,2 - 100,0	0,00	0,0 - 0,5
>10	99,79	98,8 - 100,0	10,72	8,6 - 13,2
>11	99,58	98,5 - 99,9	18,66	15,9 - 21,7
>12	99,58	98,5 - 99,9	27,86	24,6 - 31,3
>13	98,94	97,5 - 99,7	35,79	32,3 - 39,4
>14	97,88	96,1 - 99,0	44,01	40,3 - 47,7
>15	97,03	95,1 - 98,4	52,23	48,5 - 55,9
>16	95,76	93,5 - 97,4	59,33	55,6 - 62,9
>17	94,07	91,5 - 96,0	65,18	61,6 - 68,7
>18	92,16	89,4 - 94,4	69,64	66,1 - 73,0
>19	90,68	87,7 - 93,1	74,93	71,6 - 78,1
>20	88,14	84,9 - 90,9	79,39	76,2 - 82,3
>21	85,17	81,6 - 88,3	82,87	79,9 - 85,6
>22	81,36	77,5 - 84,8	84,96	82,1 - 87,5
>23	77,97	74,0 - 81,6	87,74	85,1 - 90,1
>24	73,52	69,3 - 77,4	90,39	88,0 - 92,4
>25	70,76	66,4 - 74,8	91,92	89,7 - 93,8
>26	66,31	61,9 - 70,6	93,45	91,4 - 95,2
>27	61,86	57,3 - 66,3	95,40	93,6 - 96,8
>28	54,66	50,0 - 59,2	96,24	94,6 - 97,5
>29	48,94	44,3 - 53,6	96,80	95,2 - 98,0
>30	44,70	40,2 - 49,3	98,05	96,8 - 98,9
>31	40,04	35,6 - 44,6	98,47	97,3 - 99,2
>32	35,38	31,1 - 39,9	98,89	97,8 - 99,5
>33	33,05	28,8 - 37,5	99,03	98,0 - 99,6
>34	28,81	24,8 - 33,1	99,58	98,8 - 99,9
>35	23,94	20,2 - 28,1	99,72	99,0 - 100,0
>36	20,97	17,4 - 24,9	99,72	99,0 - 100,0
>37	17,80	14,5 - 21,6	99,72	99,0 - 100,0
>38	12,92	10,0 - 16,3	99,72	99,0 - 100,0
>39	10,59	8,0 - 13,7	99,72	99,0 - 100,0
>40	7,84	5,6 - 10,6	99,72	99,0 - 100,0
>41	6,14	4,2 - 8,7	99,86	99,2 - 100,0
>42	5,08	3,3 - 7,5	99,86	99,2 - 100,0
>43	4,45	2,8 - 6,7	99,86	99,2 - 100,0
>44	2,75	1,5 - 4,7	99,86	99,2 - 100,0
>45	2,33	1,2 - 4,1	99,86	99,2 - 100,0
>46	2,12	1,0 - 3,9	100,00	99,5 - 100,0
>47	1,27	0,5 - 2,7	100,00	99,5 - 100,0
>48	0,85	0,2 - 2,2	100,00	99,5 - 100,0
>49	0,64	0,1 - 1,8	100,00	99,5 - 100,0
>50	0,00	0,0 - 0,8	100,00	99,5 - 100,0

*IC95% Intervalo de Confiança de 95% de Clopper-Pearson

## Discussão

 Esta pesquisa investigou as evidências de validade da K10, versão em português brasileiro, para rastrear o sofrimento psicológico em estudantes de nível superior, apoiando-se na estrutura interna, validade concorrente e validade preditiva. Neste estudo, os resultados, derivados do modelo de AFC Bifator, apresentaram melhores índices de ajuste em consonância com estudo anterior realizado com amostra de adultos brasileiros ^(^ 19 ^)^ . Ademais, o fator geral de sofrimento psicológico revelou elevada consistência interna e explicou percentual relevante da variância, alinhando-se a investigações anteriores que apontaram para uma estrutura unifatorial composta pelo sofrimento psicológico ^(^ 15 ^,^ 19 ^,^ 26 ^,^ 28 ^-^ 30 ^)^ . 

 Modelos bifatoriais são especialmente úteis quando se busca entender não apenas uma dimensão global, mas também variações específicas em torno dessa dimensão, oferecendo uma visão mais profunda e diversificada das relações entre as variáveis do que os demais modelos de Análise Fatorial ^(^ 38 ^-^ 41 ^)^ . Essa abordagem multifacetada é essencial para aprofundar o entendimento sobre a estrutura do sofrimento psicológico e suas manifestações variadas. 

 Nessa perspectiva, a seleção e aplicação da K10, conforme destacado por alguns autores ^(^ 19 ^,^ 29 ^)^ , devem estar intimamente ligadas ao propósito específico do instrumento e ao perfil dos indivíduos avaliados. Em contextos de populações comunitárias e processos de triagem, a detecção do sofrimento psíquico geral, em detrimento de quadros específicos, tais como ansiedade e depressão, revela-se mais conveniente. Por outro lado, no que tange à avaliação de uma população clínica, a diferenciação entre problemas específicos, como ansiedade e depressão, mostra-se mais apropriada ^(^ 29 ^)^ , o que indica a flexibilidade da K10 em atender a diferentes necessidades de avaliação. 

 A relevância dessa abordagem é especialmente significativa ao considerarmos estudantes de nível superior, nos quais a presença concomitante de sintomas ansiosos e depressivos é notável ^(^ 3 ^,^ 10 ^,^ 49 ^-^ 53 ^)^ . Esta realidade suporta a estrutura de um fator geral de sofrimento psicológico identificado neste estudo. De fato, embora o sofrimento seja frequentemente concebido, sob uma perspectiva bidimensional, como a coexistência de sintomas ansiosos e depressivos, tais quadros atuam como fatores de risco bidirecionais um para o outro ^(^ 54 ^-^ 55 ^)^ . Enquanto a ansiedade se associa ao medo, nervosismo, preocupação excessiva e sensação de tensão, a depressão relaciona-se com tristeza, desesperança, falta de interesse ou de prazer em atividades diárias, fadiga e comportamento suicida (seja por ideação suicida, tentativa ou concretização do suicídio) ^(^ 52 ^-^ 53 ^,^ 56 ^)^ . 

 Autores têm evidenciado a elevada taxa de comorbidade entre depressão e ansiedade, com impactos significativos na funcionalidade de estudantes universitários ^(^ 57 ^-^ 58 ^)^ . A sobreposição desses quadros e seus fatores de risco indicam uma relação estreita entre esses transtornos, sugerindo que sejam considerados como manifestações de uma síndrome psiquiátrica internalizante, caracterizada por sintomas voltados para o interior, tais como os sintomas ansiosos e depressivos. Essa concepção foi proposta como benéfica para a triagem e o tratamento ^(^ 59 ^)^ . A presença simultânea desses quadros intensifica o sofrimento psicológico e repercute negativamente na qualidade de vida dos estudantes ^(^ 57 ^-^ 58 ^)^ . Portanto, é crucial compreender esse fenômeno em sua totalidade e diversidade de sintomas, a fim de facilitar a elaboração e implementação de intervenções terapêuticas mais efetivas. 

 Quanto à validade concorrente, a K10 demonstrou uma elevada correlação com o SRQ-20, um dos instrumentos mais utilizados para o rastreamento de TMC. Essa performance da K10, em termos de validade concorrente, está alinhada com estudos anteriores que confirmaram essa propriedade psicométrica ^(^ 14 ^,^ 60 ^)^ . No que diz respeito ao construto avaliado, ambos os instrumentos exploram fenômenos similares, porém com suas especificidades. A K10, composta por dez itens, aborda o sofrimento psicológico por meio de sintomas depressivos e ansiosos, utilizando uma escala Likert de cinco pontos para as respostas ^(^ 14 ^)^ . Já o SRQ-20, com seus 20 itens, investiga sintomas somáticos, depressivos e ansiosos associados aos TMC, por meio de respostas dicotômicas do tipo sim/não ^(^ 17 ^-^ 18 ^)^ . 

 Em termos do *continuum* de respostas, alguns estudos ^(^ 61 ^-^ 62 ^)^ sugerem que a confiabilidade dos escores tende a aumentar à medida que se amplia o número de opções de resposta. Contudo, outros pesquisadores ^(^ 63 ^)^ não observaram melhorias significativas na precisão psicométrica de instrumentos que oferecem mais de seis alternativas de resposta. Estes apontam que escalas dicotômicas apresentam limitações quanto à consistência interna quando comparadas a formatos que disponibilizam uma gama mais ampla de escolhas. Por isso, ao selecionar um instrumento de avaliação, é recomendável considerar não apenas a diversidade de opções de resposta, mas também a facilidade de aplicação e as propriedades psicométricas validadas em diferentes amostras e estudos prévios ^(^ 63 ^)^ . 

 O SRQ-20, apesar de oferecer apenas duas opções de resposta, é amplamente reconhecido e recomendado pela OMS ^(^ 17 ^)^ devido à sua simplicidade, baixo custo e boas propriedades psicométricas. A K10, caracterizada também por sua facilidade de uso e custo acessível ^(^ 14 ^)^ , possui propriedades psicométricas robustas, as quais foram reiteradas pelos resultados deste estudo. Portanto, a escolha entre o SRQ-20 e a K10, embora ambos avaliem aspectos do sofrimento psicológico, depende essencialmente das especificidades que o pesquisador pretende explorar. Se o foco for a detecção de sintomas somáticos juntamente com sintomas depressivos e ansiosos, o SRQ-20 se mostra mais adequado. No entanto, para uma investigação mais detalhada do sofrimento psicológico sob uma ótica ampla, que abrange a variedade de sintomas depressivos e ansiosos por uma escala tipo Likert, a K10 é a opção mais indicada. 

 Nesta pesquisa, a Escala K10 sobressaiu ao apresentar uma AUC superior, em comparação aos valores encontrados em estudos anteriores, que registraram AUC de 0,90 ^(^ 16 ^)^ , 0,82 ^(^ 23 ^)^ e 0,86 ^(^ 64 ^)^ . A escala demonstrou-se precisa na versão em português brasileiro para o rastreamento de sofrimento psicológico entre estudantes universitários, alcançando elevadas taxas de sensibilidade e especificidade para pontuações acima de 21. A literatura sugere que instrumentos de rastreio devem exibir altas taxas de sensibilidade e especificidade em um ponto de corte bem definido ^(^ 48 ^)^ . Neste estudo, pontuações superiores a 21 atenderam a esses critérios, estando em consonância com os achados de pesquisas que utilizaram a versão em inglês da K10, nas quais o ponto de corte variou entre 18 a 27 pontos ^(^ 16 ^,^ 23 ^,^ 64 ^)^ . 

Embora este estudo ofereça evidências promissoras sobre a aplicabilidade da versão em português brasileiro da K10 no contexto da educação superior, é preciso considerar suas limitações, como a execução em apenas duas IFES, limitando a generalização dos resultados para outras IFES brasileiras. Sugere-se, portanto, um exame mais aprofundado das propriedades psicométricas da K10, inclusive com a aplicação da Teoria da Resposta ao Item (TRI), em amostras de diferentes regiões do país, a fim de possibilitar o contínuo refinamento desse instrumento.

Apesar dessas limitações, torna-se evidente que a estrutura global da K10, representada neste estudo por um fator geral – sofrimento psicológico, apresenta validade baseada na estrutura interna, validade concorrente e preditiva e se estabelece como um instrumento confiável para o rastreamento do sofrimento psicológico em estudantes de nível superior. Essa ferramenta pode contribuir significativamente para a elaboração e a implementação de intervenções em saúde que visam prevenir esse quadro no contexto acadêmico.

## Conclusão

Este estudo fornece evidências robustas sobre a validade da K10, sustentadas pela estrutura interna e pelas validades concorrente e preditiva, que auxiliarão no rastreamento do sofrimento psicológico em estudantes de nível superior. A AFC Bifatorial revelou ótimos índices de ajuste para esse modelo, no qual um fator geral — sofrimento psicológico — explicou a maior parte da variância dos itens da K10 e apresentou elevada fidedignidade. Além disso, a escala demonstrou validade concorrente e preditiva. Identificou-se o ponto de corte ideal da K10 para o rastreamento do sofrimento psicológico nesses estudantes, um valor em consonância com os resultados observados em investigações anteriores com a versão original.
